# Brain reactivity to emotion persists in NREM sleep and is associated with individual dream recall

**DOI:** 10.1093/texcom/tgac003

**Published:** 2022-01-27

**Authors:** Maëva Moyne, Guillaume Legendre, Luc Arnal, Samika Kumar, Virginie Sterpenich, Margitta Seeck, Didier Grandjean, Sophie Schwartz, Patrik Vuilleumier, Judith Domínguez-Borràs

**Affiliations:** 1 Campus Biotech, chemin des mines, 9 CH-1202 Geneva, Switzerland; 2 Department of Neuroscience, University of Geneva, Rue Michel Servet 1, CH-1211 Geneva, Switzerland; 3 Department of Psychology, University of Cambridge, Downing Street, Cambridge CB2 3EB, USA; 4 Department of Clinical Neuroscience, Geneva University Hospitals, 4 rue Gabrielle-Perret-Gentil 4, CH-1211 Geneva, Switzerland; 5 Department of Clinical Neuroscience, University of Geneva, 4 rue Gabrielle-Perret-Gentil 4, CH-1211 Geneva, Switzerland; 6 Department of Psychology, University of Geneva, Uni Mail, bd du Pont-d’Arve 40, CH-1211 Geneva, Switzerland; 7 Center for Affective Sciences, CISA - chemin des mines 9, CH-1202 Geneva, Switzerland

**Keywords:** brain oscillations, dream recall, emotion processing, ERP, NREM sleep

## Abstract

The waking brain efficiently detects emotional signals to promote survival. However, emotion detection during sleep is poorly understood and may be influenced by individual sleep characteristics or neural reactivity. Notably, dream recall frequency has been associated with stimulus reactivity during sleep, with enhanced stimulus-driven responses in high vs. low recallers. Using electroencephalography (EEG), we characterized the neural responses of healthy individuals to emotional, neutral voices, and control stimuli, both during wakefulness and NREM sleep. Then, we tested how these responses varied with individual dream recall frequency. Event-related potentials (ERPs) differed for emotional vs. neutral voices, both in wakefulness and NREM. Likewise, EEG arousals (sleep perturbations) increased selectively after the emotional voices, indicating emotion reactivity. Interestingly, sleep ERP amplitude and arousals after emotional voices increased linearly with participants’ dream recall frequency. Similar correlations with dream recall were observed for beta and sigma responses, but not for theta. In contrast, dream recall correlations were absent for neutral or control stimuli. Our results reveal that brain reactivity to affective salience is preserved during NREM and is selectively associated to individual memory for dreams. Our findings also suggest that emotion-specific reactivity during sleep, and not generalized alertness, may contribute to the encoding/retrieval of dreams.

## Introduction

The human brain has evolved to efficiently detect and respond to emotional signals in order to promote survival (Vuilleumier 2005; Domínguez-Borràs and Vuilleumier 2013). In the auditory domain, emotional vocalizations constitute a privileged way of communicating imminent danger across conspecifics (Sauter et al. 2010). These types of stimuli, together with other nonvocal sounds (e.g. gunshots), elicit increased responses in the temporal auditory cortex when compared with neutral stimulation (Belin et al. 2004; Grandjean et al. 2005; Wiethoff et al. 2008; Ethofer et al. 2012; Fruhholz et al. 2012; Arnal et al. 2015). Emotion detection in sounds is also reflected in the modulations of the event-related potential (ERP) responses, with differential effects starting ~100–200 ms after stimulus-onset (De Baene et al. 2004; Goydke et al. 2004; Schirmer et al. 2004, 2005; Sauter and Eimer 2010; Liu et al. 2012; Hung and Cheng 2014; Pannese et al. 2015), or in modulations of oscillatory neural activity in specific frequency ranges, such as in the delta, theta, beta, or gamma bands (Symons et al. 2016; Blume et al. 2017). The limbic system, including the amygdala, is believed to play a pivotal role in such amplification of sensory responses (Vuilleumier 2005; Adolphs 2008; see Pannese et al. 2015 for review).

In states of reduced responsiveness such as during sleep (Ludwig 1966; Hobson and Pace-Schott 2002), emotion reactivity may become most critical in order to ensure prompt detection of potential threats (Kirov et al. 2012). However, the extent to which this ability remains functional in conditions of limited awareness is debated, even in wakefulness (Vuilleumier et al. 2001; Pessoa et al. 2002; Whalen et al. 2004; Pessoa and Adolphs 2010; Tamietto and de Gelder 2010; see Dominguez-Borras et al. 2012). During sleep, humans undergo progressive disconnection from external stimulation as they transition toward deeper stages (Jouvet 1967a), ensuring physiological continuity (Lecci et al. 2017). This disconnection is reflected by an increase in arousal threshold, measured as the difficulty to wake up after sensory stimulation, including sounds (Jouvet 1967b). Such a decrease in stimulus-responsivity seems more stable during non-REM (NREM) than during rapid eye movement (REM) sleep, where the level of alertness may be more variable through time (Sallinen et al. 1996; Atienza et al. 2001).

However, despite a progressive disengagement from the external sensory environment during NREM, the brain is still able to detect and differentially respond to specific salient stimuli, such as deviant or novel sounds, familiar voices, or the subject’s own name (see Perrin et al. 1999; Pratt et al. 1999; Portas et al. 2000; Atienza et al. 2001, 2002; Cote 2002; Ruby et al. 2008; Strauss et al. 2015; Blume et al. 2018; Legendre et al. 2019). Similarly, mothers are more easily awoken by their own infants’ cries than by other babies’ cries (see Formby 1967; Ruby 2011). These observations suggest that differential processing of affective signals may still occur during deep sleep. Accordingly, high levels of activity/metabolism have been reported in several emotion-related regions, such as the ventral striatum, anterior cingulate cortex, or amygdala, during NREM compared with waking state (Nofzinger et al. 2002). Moreover, NREM sleep is characterized by sleep spindles in electroencephalography (EEG), which have also been associated to the activation of emotion-responsive regions (i.e. anterior cingulate and insula; Schabus et al. 2007). Finally, one study reported that, during the lighter phase 2 of NREM sleep, names pronounced with an angry voice can evoke increased delta and sigma responses in EEG (the latter possibly associated to spindle activity), compared with voices with neutral prosody (Blume et al. 2017). This suggests that verbal stimuli with emotional prosody may be detected as distinctively salient, at least during lighter NREM sleep.

On the other hand, brain reactivity to external stimulation in sleep may be highly variable due to various physiological factors, not only across sleep stages (e.g. Andrillon et al. 2016; Lecci et al. 2017; Legendre et al. 2019) but also across individuals. Individual variables, such as anxiety traits, are known to influence emotion detection or sensory responses more generally (Mogg and Bradley 2002; Rossignol et al. 2012). Another feature varying with individual stimulus reactivity is dream recall frequency. Several studies reported that high dream recallers (individuals who can frequently report their dreams over a period of time) show increased electrophysiological responses to auditory inputs, compared with low dream recallers (Ruby et al. 2013b; Eichenlaub, Bertrand, et al. 2014; Vallat et al. 2017). Accordingly, it has been suggested that the brain of high dream recallers might be more reactive toward external stimulation, making these individuals more prone to brief awakenings, ^*^^*^a characteristic that would favor memory encoding of dream content (Cohen 1972; Ruby et al. 2013b; Eichenlaub, Bertrand, et al. 2014; Eichenlaub, Nicolas, et al. 2014; Vallat et al. 2017). In fact, dream recall frequency is known to increase with the number of awakenings during sleep (Cory and Ormiston 1975; Schredl et al. 2003; Ruby 2011). Altogether, these observations converge to suggest that dream recall frequency may provide a reliable marker of individual cortical reactivity during sleep. However, to date, there is no direct evidence supporting systematic associations between this feature and individual neural responsiveness. Moreover, it is unknown whether dream recall is associated with generalized alertness toward sensory stimulation or might reflect more specific processing biases for stimulus salience or affective value. This would be plausible given that dream reports often contain strong emotions (Desseilles et al. 2011; Ruby 2011; Sterpenich et al. 2020), a phenomenon that seems closely related to limbic brain activity (Perogamvros and Schwartz 2012, 2015; Blake et al. 2019). This would imply that high dream recallers possess a particular bias toward affective processes, with stronger production, encoding, or recollection of dreams, and, relatedly, increased reactivity toward affective signals.

In the present study, we aimed at characterizing how the NREM brain responds to emotion across lighter and deeper sleep stages, and whether such responses vary according to individual dream recall frequency. Specifically, we investigated EEG responses (ERPs and oscillatory activity) evoked by meaningless vocal utterances with either affective or neutral prosody, both during wakefulness and NREM sleep in 13 healthy young participants. Then, to further characterize neural reactivity during sleep, we carefully examined sleep EEG arousals, indicating subtle (but not full) awakenings (Iber et al. 2007), occurring after each stimulus category. Finally, we tested whether these neural responses would vary linearly with dream recall frequency of participants. Dream recall frequency was calculated as the total number of dreams remembered over 7 consecutive nights prior to, and during, the night of the experiment, as reported in a dream diary by the participants. First, we predicted that a differential brain response to emotion (vs. neutral) would persist in NREM sleep, despite fading levels of consciousness. Based on previous literature, increased neural reactivity to emotional sounds should be reflected by larger ERP amplitudes (Sauter and Eimer 2010; Liu et al. 2012; Hung and Cheng 2014) and increased spectral power along different frequency bands (e.g. Guntekin and Basar 2007; Balconi and Pozzoli 2009; Ruby et al. 2013a, 2013b). Preserved emotion reactivity during sleep might also lead to more frequent arousals (relative to neutral sounds), reflecting subtle sleep perturbations induced by the affective stimulation. Second, we hypothesized that stimulus reactivity in sleep would increase linearly with dream recall frequency, as stronger susceptibility to sleep perturbations is believed to favor memory encoding of dreams (Ruby et al. 2013b; Eichenlaub, Bertrand, et al. 2014). Furthermore, if dream recall is associated with generalized stimulus reactivity, as previously suggested, neural responsivity should be stronger as dream recall frequency increases regardless of stimulus type (i.e. similarly for neutral and emotional stimuli). However, if dream recall is associated with selective reactivity biases toward affective salience, such increase should be specific for emotional voices. This would in turn support the importance of affective processes in dreaming, as previously postulated (Perogamvros and Schwartz 2012), and shed new light on mechanisms mediating dream memory encoding and recall.

## Materials and methods

### Participants

We recruited a total sample of 23 healthy, normal hearing, right-handed young adults. Six participants were not considered for final analysis due to poor signal quality (*n* = 3) or bad sleep conditions (*n* = 3). From this sample, we selected only participants who displayed reliable EEG signal both during the AWAKE and the SLEEP sessions. This yielded a final group of 13 participants (8 females), aged 19–24 years (M = 21.69, SD = 1.6), with no past neurological or psychiatric history, no reported specific phobias, drug consumption, or abnormal audition. All participants were considered good sleepers, after thorough screening with specific questionnaires (see Table 1) and standard evaluation of medical aspects and sleep quality. We also assessed anxiety levels using the State–Trait Anxiety Inventory (STAI-T; Spielberger et al. 1983) and the Beck Anxiety Inventory (BAI; Beck et al. 1988), showing overall scores within the normal range (Table 1). Finally, all participants gave written informed consent, and the experimental protocol was approved by the local Ethical Committee of the University of Geneva, in accordance with the Declaration of Helsinki.

**Table 1:** Participants’ demographic data and questionnaire scores.

Subject	Gsender	Age	Dream recall frequency (HR/LR)	Beck Depression Inventory (BDI)^a^	Beck Anxiety Inventory (BAI)^a^	Epworth sleepiness scale (ESS)^a^	STAIT-T trait-anxiety test^b^	Circadian typology Horne and Osberg^c^	Edinburgh laterality test^d^	PSQI-1 sleeping habits test^a^	Consumption habits (AUDIT)^a^
S1	F	23	0 (LR)	5^*^	4^*^	7^*^	26^*^	59 M^*^	16 R	3^*^	7^*^
S2	M	24	6 (HR)	1^*^	0^*^	4,5^*^	32^*^	51 -	23 R	4^*^	0^*^
S3	F	20	2 (LR)	8^*^	4^*^	6^*^	42^*^^*^	65 M^*^	60 R	3^*^	0^*^
S4	F	21	6 (HR)	3^*^	2^*^	3^*^	30^*^	47 -	17 R	1^*^	2^*^
S5	F	22	1 (LR)	4^*^	3^*^	3^*^	26^*^	47 -	11 R	5^*^	7^*^
S6	F	19	2 (LR)	0^*^	0^*^	4^*^	25^*^	58 -	22 R	1^*^	7^*^
S7	F	20	3 (LR)	3^*^	1^*^	12^*^^*^	35^*^	57 M^*^	20 R	5^*^	5^*^
S8	F	23	6 (HR)	2^*^	2^*^	2^*^	49^*^^*^	45 -	17 R	2^*^	3^*^
S9	M	24	6 (HR)	2^*^	3^*^	2^*^	35^*^	43 -	24 R	6^*^	1^*^
S10	M	20	1 (LR)	2^*^	0^*^	2.5^*^	35^*^	32 -	17 R	2^*^	1^*^
S11	M	22	4 (HR)	0^*^	1^*^	8.5^*^	26^*^	65 M^*^	24 R	3^*^	5^*^
S12	M	22	2 (LR)	0^*^	0^*^	3^*^	21^*^	57 -	20 R	2^*^	3^*^
S13	F	22	1 (LR)	0^*^	0^*^	6^*^	24^*^	43 -	18 R	1^*^	7^*^
Mean	--	21.69	3.1	2.31	1.54	4.88	31.23	51.46	22.23	2.92	3.69
Std Dev	--	1.6	2.25	2.36	1.56	2.95	7.93	9.71	11.93	1.66	2.78

Dream recall is indicated as the number of nights (over a period of 8 consecutive nights) with at least one dream reported. HR and LR stand for high and low dream recaller, respectively. Std Dev stands for standard deviation.

^a^^*^normal/^*^^*^moderated.

^b^^*^low/^*^^*^moderated.

^c^M, morning/^*^moderated/- neither morning nor evening.

^d^R, right.

### Stimuli

Stimuli were binaural meaningless but word-like vocal utterances pronounced with either angry (NEG condition; 6 stimuli) or neutral prosody (NEU condition, 6 stimuli), selected from a previously validated database (Banse and Scherer 1996), and used elsewhere (Grandjean et al. 2005). Stimuli were performed by 6 different actors (3 females), each pronouncing the same pseudo-word (meaningless syllable sequences “goster” and “figotleich”) with angry or neutral prosody. Sounds were equated for mean sound-pressure level (70-dB SPL), and for average energy in all frequencies within the sound spectrum, resulting in equal perceived loudness. Stimulus duration was 750 ms. All sounds were delivered through Sennheiser®3.00 Cx in-ear headphones, fixated with tape onto the ears, to avoid removal during sleep. An extra set of control voices (6 ControlNEG and 6 ControlNEU stimuli), with the same envelope as the experimental voices but filled with white noise with the software Praat (Boersma and Weenink 2018), was also included.

### Procedure and stimulus delivery

Participants came twice to the Sleep Laboratory at the Clinical and Sleep Research Unit of Campus Biotech (https://www.campusbiotech.ch/en/) for two different sessions: first, a habituation night during which participants familiarized with the experimental settings, by spending one whole night in bed under similar conditions to those they would have during the experimental night. Participants arrived at the laboratory about 2 h before their habitual evening bedtime. They were given all the necessary explanations, signed the consent form, and filled in the St. Mary’s Hospital Questionnaires with other brief evaluations to ensure that sleep quality on the previous night, consumption of any stimulants, as well as physical and emotional self-reported states, were appropriate according to standard criteria. Participants were given a dream diary, which they filled in every morning for 1 week, starting from the morning after the habituation night until the morning after the experimental night (8 daily reports in total). They also received an actimeter and had to wear it continuously, day and night, until the experimental night (included), to verify that the 4 nights of sleep prior to the experimental night were of good quality. Participants then got prepared for the polysomnography recording, with their sleepwear on. At a time corresponding approximately to participants’ habitual bedtime, the experimenters switched the light off (i.e. onset for sleep latency measurement). No tasks were performed during the habituation session, and participants wore no headphones. On the next morning, participants were awoken by the experimenters (after about 8 h of sleep), showered, and had breakfast.

**Fig. 1:**
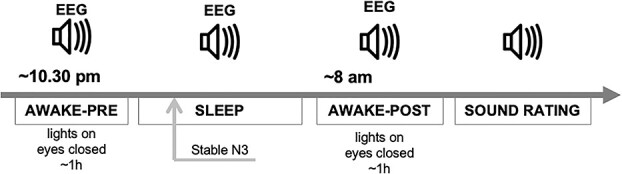
Procedure during the experimental night. Participants were presented with voices before going to sleep for about 1 h (AWAKE-PRE). During sleep (SLEEP), voices were first delivered as soon as NREM-stage 3 (N3) was detected, and were continuously presented during the first ~3 h and the last ~2 h of the night. In the morning, participants were awakened and again presented with the voices for about 1 h (AWAKE-POST). At the end of the experiment, participants rated the voices that had been presented before.

The experimental night took place 1 week later, and the overall procedure was almost identical to the habituation night, with the exception that sounds were volume-adjusted and delivered overnight. Thus, after preparation for polysomnography, participants sat down comfortably on a chair with headphones on and eyes closed and performed a short, standardized test to assess their hearing threshold. Sounds would then be delivered at an intensity of 30% above hearing threshold for all experimental sessions (except for 2 participants, for whom volume had to be lowered during the sleep sessions due to frequent awakenings, see below for further details). Then, participants underwent the first part of the experimental session (the AWAKE-PRE session; Fig. 1), while still seated (keeping eyes closed to avoid distraction by visual stimulation). The task consisted of a passive listening of sounds, delivered every 8 (±3) s with the software *Cogent 2000* v1.32 (Wellcome Department of Imaging Neuroscience, London, UK) running on Matlab 2012*.* Stimuli could be from either the NEU or the NEG conditions (or from the corresponding control), delivered in a pseudo-randomized sequence, so that stimuli from the same condition would never be delivered more than twice in immediate succession. The AWAKE-PRE session lasted about 1 h. To avoid excessive drowsiness during this session, an experimenter entered the room every 15 min (or whenever drowsiness was detected from the control room) to check on the participant, reiterate instructions, and give information about the remaining time.

Then, participants went to bed, and the experimenters monitored their polysomnography from a control room. As soon as stage 3 of NREM sleep (N3) was detected (i.e. after 2 min of continuous well-defined slow waves), the SLEEP session started (Fig. 1) and sounds were delivered throughout the night until awakening, with the exact same presentation parameters as during the AWAKE-PRE session. We chose not to launch the task during the first cycle of lighter sleep stages (1 or 2), given that starting to play sounds during that period would have resulted in more probable awakenings (Rechtschaffen et al. 1966) and thus data loss. If a full awakening was detected during the night (i.e. observable EEG perturbations during more than 15 s; Iber et al. 2007), the sequence was stopped and then resumed once stable N3 sleep stage was reached again. Participants were monitored with a real-time infrared video system throughout the night. Since a typical night is richer in slow wave sleep during the first hours and in REM sleep during the last hours (Carskadon and Dement 2011), and we were initially aiming for a balanced number of trials across sleep stages, sounds were mainly delivered during the first ~3 h of the night and during the last ~2 h of the night. However, note that REM sleep was ultimately excluded from analysis due to an insufficient number of clean trials. This was mainly due to emotional sounds leading to frequent awakenings during REM sleep. In the morning following the experimental night, participants were awoken by the experimenters after about 8 h of sleep, and then again presented with the same sounds (AWAKE-POST session) and with the exact same characteristics and procedure as in the initial baseline (AWAKE-PRE session). Finally, participants rated all sounds they heard during the experimental sessions on a scale from −5 to 5 (including the neutral zero value), for both arousal (being −5 the lowest value and 5 the highest value) and emotional valence (being −5 the most negative valence and 5 the most positive valence). Sounds were played in random order, with the exact same volume as the one used throughout the experiment. Again, note that for both participants for whom volume had to be lowered during the night, this lowered sound volume was kept for the AWAKE-POST session and sound ratings. Before leaving the laboratory, participants showered, had breakfast, filled in their dream diary for the last time, and were paid 250 CHF.

### Polysomnography

Electroencephalogram (EEG), electrooculogram (EOG), and electromyogram (EMG) were recorded continuously during all sessions with Brain Vision Recorder© using a BrainAmp Standard Amplifier (Brainproducts Inc., Gilching, Germany) at a 500-Hz sampling rate. During the experimental sessions, the EEG was recorded from 12 scalp sites (F3, Fz, F4, C3, Cz, C4, T3, T4, P3, Pz, P4, and Oz), according to the 10/20 system (Jasper 1958). To improve electric conductance, dead skin and sebum excess were eliminated from the scalp and face with NuPREP® skin Prep Gel (Weaver and Company) and electrodes were attached to the scalp with EC2® Electrode Cream (Natus®Neurology). The horizontal and vertical EOG were recorded with electrodes placed above the right outer canthus and below the left outer canthus, respectively. In turn, EMG was recorded with bipolar submental electrodes. An electrode placed on the middle of the forefront was used as a common reference and ground was located on the left outer canthus. Impedances were kept below 10 kΩ. Galvanic skin response and heartbeat were also recorded but will not be presented here.

Sleep scoring of all sessions (AWAKE and SLEEP) was conducted offline by two independent and experienced raters, after rereferencing the EEG signal to the average of electrodes T3 and T4, with the FASST® toolbox run on Matlab R2009b, following the criteria of the American Academy of Sleep Medicine (Iber et al. 2007; Berry et al. 2012). Note that this rereferencing was only used for EEG visualization during scoring. Interscorer agreement was above 90%. In case of doubt or scoring ambiguity, trials were excluded from analysis. The continuous EEG was classified as either wakefulness (W), stage 1 (N1), stage 2 (N2), stage 3 (N3), or Rapid-Eye Movement (REM) sleep using 20-s epochs. Epochs with large blinks or movement artifacts were already eliminated with FASST® from further analysis, as a prior step before artifact rejection during signal preprocessing. Arousals were also identified with FASST® whenever there was a short perturbation in the EEG signal, indicating short awakenings (Iber et al. 2007; see the NREM arousals section).

### Dream recall

We hypothesized that dream recall frequency might reflect cortical reactivity during sleep and thus be influenced by the processing of sounds during sleep (Ruby et al. 2013b; Eichenlaub, Bertrand, et al. 2014; Eichenlaub, Nicolas, et al. 2014; Vallat et al. 2017). To assess dream recall frequency, we examined dream reports from the participants’ diaries over the 8 consecutive nights (Table 1). Dream recall frequency was calculated as the total number of nights from which at least one dream would be reported (regardless of the number of dreams recalled per night; Ruby et al. 2013b ; Eichenlaub, Bertrand, et al. 2014 ; Vallat et al. 2017). For consistency with previous literature (Ruby et al. 2013b; Eichenlaub, Bertrand, et al. 2014; Vallat et al. 2017), we split our sample into two groups, where participants reporting dreams after 4 nights or more (i.e. 50% of the total record of nights) were considered high recallers (HRs; *n* = 5; mean dreams reported: 5.60; SD = 0.89), while the others were low recallers (LR; *n* = 8; mean dreams reported: 1.50; SD = 0.92). Note that our criteria to separate both groups differed from the above-mentioned studies where subjects were classified based on prescreening questionnaires and considered either HRs or LRs upon confirming dream recall on >3 mornings per week or <2 mornings per month, respectively. Instead, we used a direct, prospective quantification of dream recall for the nights preceding, and immediately following, the experimental session (as well as a complete verbal record of dream content, not reported here). Finally, to bolster the robustness of our findings (considering the small size of HR and LR groups), we also performed Pearson’s correlations between dream recall frequency and EEG data of interest (see next sections). *P*-values of these correlations were one-tailed and false-discovery-rate (FDR) corrected to control for type I errors, unless stated otherwise.

### Voice ratings

Mean voice ratings for emotional valence and arousal, obtained from each participant in the morning following the experimental night, were also analyzed. Due to technical problems, ratings from four participants (Subjects 1, 3, 10, and 11) were lost. Valence and arousal scores from the 9 remaining participants were tested with two-way mixed ANOVAs (with Greenhouse–Geisser adjustments to the degrees of freedom), comprising the within-subjects factor Emotion (NEG, NEU) and, to rule out any possible individual biases in ratings, the between-subjects factor Dream recall (HR, LR).

### E‌EG preprocessing

EEG preprocessing was carried out using custom-written scripts for the toolbox Fieldtrip (Oostenveld et al. 2011) implemented in Matlab (Mathworks, R2015b). Independent-component analysis was performed to identify eye-blink and eye-movement components, which were removed from the EEG signal. Furthermore, channels from individual subject data with apparent low signal-to-noise ratio after visual inspection were removed and their signal was interpolated from neighboring channels. EEG data were then segmented into epochs from −1000 to 2000 ms around the onset of the auditory stimuli, and band-pass filtered from 0.5 to 30 Hz (for ERP analysis), or high-pass filtered with a 0.5-Hz cutoff (for time–frequency analysis). Data were downsampled to 100 Hz (for ERP analysis) and to 200 Hz (for time–frequency analysis) to reduce computation time. Subsequently, trials were sorted by conditions and sleep stages (the latter according to sleep scoring). Then, data were z-scored for baseline correction, using the prestimulus (−200 to 0 ms) average and standard deviations across trials and conditions. This approach was chosen to overcome large variabilities in the EEG signal over time (e.g. Makeig et al. 2004). ERPs were averaged for each condition and participant over a peristimulus epoch of −200 to 1000 ms. Finally, the grand average across all participants was computed for each condition and sleep stage.

### ERP analysis

Since memory consolidation or habituation effects related to emotional stimulation were out of the scope of present study, we considered that both the AWAKE-PRE and AWAKE-POST sessions pooled together for our analyses. We also focused on sleep stages N2 and N3 of NREM, as these were the most represented stages in our data, and given that N1 is characterized by frequent behavioral (conscious) responsiveness from the sleeper to external stimulation (Campbell 2010). In addition, given that some participants had few clean trials in N2 or N3 and that we did not aim at differentiating stimulus-driven responses between these two stages, our NREM data combined trials from both N2 and N3. In the AWAKE (PRE and POST) condition, the average number of trials retained for analysis was 75.4 (min: 44; max: 116) for NEU, 82.7 (min: 45; max: 126) for NEG, 82.8 (min: 42; max: 114) for ControlNEU, and 77.1 (min: 48; max: 117) for ControlNEG. For the NREM condition, the average number of trials retained for analysis was 81.5 (min: 49; max: 145) for NEU, 97.4 (min: 68; max: 153) for NEG, 94.2 (min: 66; max: 149) for ControlNEU, and 83.8 (min: 53; max: 136) for ControlNEG (see Supplementary Material for further details).

Lastly, to control for any possible confounds related to unequal proportion of N2 vs. N3 trials between HRs and LRs, and thus ensure that Dream recall frequency effects did not simply reflect lighter sleep for HRs, we ran additional control analyses detailed in the Supplementary Material.

To examine emotion-related neural responses during wakefulness and NREM sleep, we extracted the mean voltage values from the averaged data across four consecutive time-windows (see next) for each brain state, condition, and channel, and submitted these data to repeated-measure ANOVAs. We included the factors State (AWAKE, NREM), Emotion (NEU, NEG), Time-Window (0–200 ms, 200–400 ms, 400–600 ms, 600–800 ms), Frontality (F3, Fz, F4 vs. T3, Cz, T4 vs. P3, Pz, P4), and Laterality (F3, T3, P3 vs. Fz, Cz, Pz vs. F4, T4, P4). To simplify the model, we only tested the conditions NEG and NEU, excluding the control sounds, which were evaluated in separate ANOVAs (see below).

Then, to further test the hypothesis that stimulus reactivity in high vs. low dream recallers may impact affective processing of sounds during sleep (Ruby et al. 2013b; Eichenlaub, Bertrand, et al. 2014; Vallat et al. 2017), we included a between-subject factor Dream recall (LR, HR). Because we hypothesized that the number of EEG arousals (see below) occurring after sounds during sleep may be linked to individual stimulus reactivity and thus directly modulate individual dream recall frequency (Vallat et al. 2017), we also included a covariate representing the average number of EEG arousals occurring after all stimulus conditions together. Please note that this was considered relevant despite the fact that trials containing arousals during NREM were excluded from the ERP analysis, as a result of signal preprocessing. In addition, given that anxiety levels may influence emotional detection (Mogg and Bradley 2002; Rossignol et al. 2012), we also included individual Beck anxiety scores as a covariate. We considered this scale as being the most representative of recent experienced anxiety levels.

For all ANOVAs, Greenhouse–Geisser adjustments to the degrees of freedom were used. Again, given our sample size, partial eta squared and observed power estimates are provided in key tests for a 0.05 alpha level. Post-hoc tests were conducted whenever there were significant interactions between the main factors (Bonferroni-corrected, unless stated otherwise). Furthermore, independent sample *t*-tests were conducted to compare voltage values across HRs and LRs, and conditions. Note that we used here parametric tests given that the voltage values were normally distributed (as confirmed by Shapiro–Wilk tests) for all conditions and Dream recall groups.

Finally, to rule out that the observed Emotion effects would stem from physical features and not from the affective content of the sounds (Grandjean et al. 2005), we implemented the same analyses but this time considering only the ControlNEU and ControlNEG conditions. Conclusively, to further explore any observed significant effects of Dream recall on stimulus- or emotion-responses, we performed again bivariate Pearson’s correlations (FDR-corrected, unless stated otherwise) between Dream recall frequency and mean voltage values for the NEU, NEG, ControlNEU, and ControlNEG conditions and for time-windows of interest.

### Analysis of oscillatory activity

Stimulus-related oscillatory responses were analyzed with Fieldtrip. After signal preprocessing (see above), single trials were decomposed into their frequency components and then averaged for each condition and participant. Time–frequency transforms were carried out with complex-valued Morlet wavelets. To limit the influence of window length on resulting time courses, we used time-windows of decreasing size with increasing frequency (Staresina et al. 2015). We used logarithmically increasing full number of cycles from 2 to 10 cycles for frequencies spanning from 1 to 100 Hz, therefore resulting into windows from 2^*^1 to 10^*^100 ms. The analysis window was centered on −0.8 to 2 s, sliding in steps of 0.005 s. A baseline correction (−500 to −100 ms before stimulus onset) was implemented by means of decibel conversion, and grand averages of all participants were calculated for each condition (NEU, NEG, ControlNEU, and ControlNEG) and state (AWAKE and NREM). Statistics were computed by means of cluster-based permutation tests, with 1000 randomizations.

First, we evaluated emotion-related responses for normal sounds (NEU vs. NEG) and control sounds (ControlNEU vs. ControlNEG), across all participants, with dependent-sample *t*-tests (two-tailed). Second, we compared stimulus-responses in HRs vs. LRs, with independent sample *t*-tests (two-tailed). For that, we first compared responses to each condition separately (NEU, NEG, ControlNEU, and ControlNEG) with the between-subject factor Dream recall (HR vs. LR). In turn, emotion response (NEU vs. NEG) was also compared across groups with a subtraction method, where differences between NEG and NEU for each subject were tested with the between-subject factor Dream recall. The statistic in all cases was calculated over a time-window from 0 to 1000 ms after stimulus-onset and including the same electrodes as those included in the ERP analysis (F3, Fz, F4, T3, Cz, T4, P3, Pz, and P4).

We analyzed different frequency bands of interest, based on previous literature on brain oscillations associated to emotion and stimulus reactivity. These included theta (4–8 Hz; Balconi and Lucchiari 2006; Balconi and Pozzoli 2009), alpha (8–12 Hz; Guntekin and Basar 2007; Del Zotto et al. 2013; Ruby et al. 2013b), beta (12–30 Hz; Schutter et al. 2001; Guntekin and Basar 2007; Guntekin and Tulay 2014), and gamma (30–80 Hz; Balconi and Pozzoli 2009). Furthermore, and specifically for the NREM stage, delta (0.5–4 Hz; Knyazev 2012; Blume et al. 2017) and sigma (11–15 Hz) frequency bands were also examined, the latter reflecting stimulus-associated spindles in NREM, which have been linked to increased activity in emotion-related regions (Schabus et al. 2007). Clusters were identified based on temporal, spatial, and spectral adjacency and were only retained when reaching significance (with an overall threshold of *P* = 0.05) after cluster-correction.

Finally, and again to further explore any observed significant effects of Dream recall on stimulus- or emotion-associated responses, we performed once more Pearson’s bivariate correlations (FDR-corrected, unless stated otherwise) between Dream recall frequency and mean voltage for each condition (NEU, NEG, ControlNEU, and ControlNEG) over the channels, time-windows, and frequency bands of interest.

### NREM arousals

Finally, to further characterize neural reactivity to sounds, we examined EEG arousals during NREM. These were classified according to sleep scoring and extracted for quantification with Matlab. Thus, arousals were identified as shifts in EEG frequency to alpha, theta, or frequencies greater than 16 Hz (but not spindles), for a minimum of 3 s and a maximum of 15 s, with at least 10 s of stable sleep preceding the change (Iber et al. 2007). We restricted our analysis to arousals given that they are more frequent than full awakenings (Bastuji et al. 2008), and given that, whenever awakenings were detected during the night, the experimental sequence was interrupted to avoid EEG data loss. Furthermore, from the identified arousals, we considered for analysis only those starting 0–4 s after sound onset, not before or later. This criterion was stricter than that implemented in previous research, which typically considered arousals as stimulus-related if they occurred within 15-s poststimulus (Bastuji et al. 2008; Vallat et al. 2017; American Sleep Disorders Association 1992). With this strategy, we also aimed at ensuring that arousals would be most likely evoked by the immediately preceding sound and not by earlier stimulation. Then, we extracted the number of arousals that occurred after sound presentation for each condition and compared the corresponding means using a repeated-measure ANOVA, with the software IBM SPSS Statistics (version 25). This ANOVA included the within-subject factors Emotion (NEG vs. NEU) and Sound type (Normal sound, Control sound), and the between-subject factor Dream recall (HR, LR). Furthermore, for descriptive purposes, and to better specify the patterns of occurrence of these arousals with respect to the preceding sounds, we also examined arousal onset times with respect to the onset of the preceding sound (see Supplementary Material). For all ANOVAs, Greenhouse–Geisser adjustments to the degrees of freedom were used. Given our sample size, partial eta squared values are provided, and post-hoc power analyses were conducted on key comparisons with the software G^*^power, for a 0.05 alpha level (Faul et al. 2007). Post-hoc tests were conducted whenever there were significant interactions between the main factors. In turn, paired-wise comparisons were computed for each Dream recall group separately, using signed-rank nonparametric tests (Wilcoxon). Finally, we examined whether a linear relationship existed between individual dream recall frequency and the number of arousals occurring after the NEU, NEG, ControlNEU, and ControlNEG sounds separately, again by means of Pearson’s correlations.

## Results

### Voice Ratings

Mean ratings of valence (emotional positivity vs. negativity) across participants were 0.47 for the NEU and −1.77 for the NEG voices (on a scale from −5 to 5). Mean ratings of voice arousal were −1.37 for NEU and 1.26 for NEG. Thus, angry voices were rated as being more negative and more alerting than neutral voices (Valence: *F*_(1,7)_ = 39.47; *P* = 0.00041; Arousal: *F*_(1,7)_ = 83.84; *P* = 0.000038), supporting the validity of our stimuli in terms of emotional content (Banse and Scherer 1996; Grandjean et al. 2005). There were no significant between-subject effects of the factor Dream recall for either valence (*P* = 0.9) or arousal (*P* = 0.32) nor Emotion × Dream recall interactions (*P* > 0.4), indicating no consistent biases in ratings among participants with different dream recall frequencies (HR vs. LR).

### Event-related Potentials

**Fig. 2:**
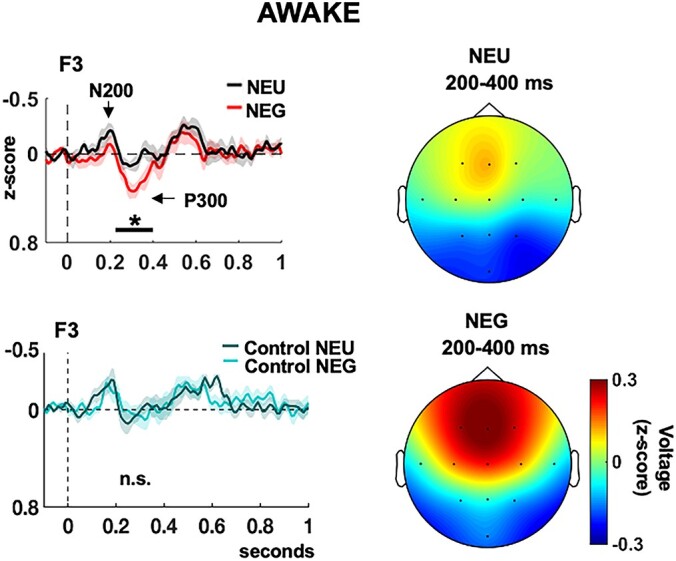
(Top left) ERPs to neutral (NEU) and angry (NEG) voices during wakefulness for all participants, showing emotional modulation. (Bottom left) ERPs to control sounds, showing no differences among conditions. (Right) Voltage distribution of the significant window for NEU and NEG voices. Shaded areas indicate the standard error of the mean (s.e.m.). ^*^P < 0.05.

**Fig. 3:**
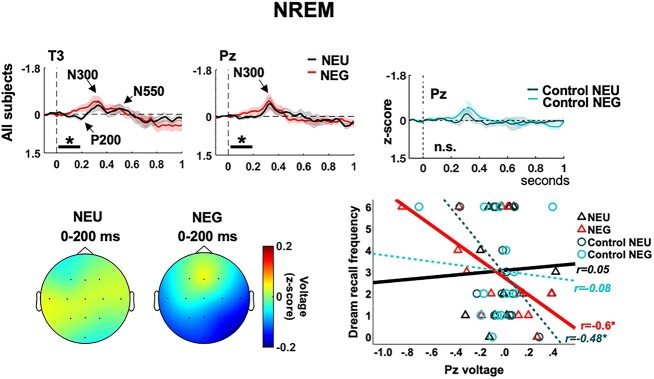
ERPs to neutral (NEU) and angry (NEG) voices during NREM for all participants. (Bottom left) Voltage distribution of the significant window for NEU and NEG voices. (Top right) ERPs to control sounds, showing no significant differences. (Bottom right) Scatter plots and lines of best fit illustrating voltage values for the significant time-window in Pz after each sound condition vs. participants’ dream recall frequency. Pz voltage values for the emotional voices correlated strongly with individual dream recall. Shaded areas indicate the standard error of the mean (s.e.m.). ^*^P < 0.05.

A summary of ERP statistics is reported in Table 2. A first omnibus ANOVA comparing ERPs time-locked to the voices (NEU and NEG), for both AWAKE and NREM states, and for all four consecutive 200-ms windows poststimulus onset, yielded significant differences between states. This indicates an (expected) difference in auditory responses across sleep and wake states (Atienza et al. 2001; Figs 2 and 3). This ANOVA also revealed a group difference between the HR and LR participants, but depending on brain state (Table 2; State × Dream recall interaction, no main effect of Dream recall). Whereas ERPs during wakefulness were similar for both groups, they showed larger amplitudes for HRs than LRs during NREM (Supplementary Fig. 1a). This group difference was more pronounced on centro-parietal sites and for specific time-window(s), as reflected by a significant State × Time-Window × Frontality × Dream recall interaction (Table 2).

**Table 2:** Summary of ERPs statistics (ANOVAs and *t*-tests).

	Awake				NREM			
	Win1	Win2	Win3	Win4	Win1	Win2	Win3	Win4
State	*F*_(1,9)_ = 5.41; *P* = 0.045^*^; η*_p_*^2^ = 0.38							
State × Time-Window × Emotion	*F*_(3,27)_ = 4.42; *P* = 0.018^*^; η*_p_*^2^ = 0.33; power [1 − β err prob]: 0.61							
State × Dream recall	*F*_(1,9)_ = 20.25; *P* = 0.001^*^^*^; η*_p_*^2^ = 0.98							
State × Time-Window × Frontality × Dream recall	*F*_(6,54)_ = 5.51; *P* = 0.017^*^; η*_p_*^2^ = 0.38							
Emotion × Dream recall	*P* > 0.1	*P* > 0.1	*P* > 0.2	*P* > 0.2	*F*_(1,9)_ = 6.13; *P* = 0.035^*^; η*_p_*^2^ = 0.41; power [1 − β err prob]: 0.6	*P* > 0.05	*P* > 0.1	*P* > 0.8
Emotion in high recallers	--	--	--	--	*F*_(1,2)_ = 43.52; *P* = 0.022^*^; η*_p_*^2^ = 0.96; power [1 − β err prob]: 0.89	--	--	--
Emotion in low recallers	--	--	--	--	*F*_(1,5)_ = 9.6; *P* = 0.027^*^; η*_p_*^2^ = 0.66; power [1 − β err prob]: 0.69	--	--	--
High vs. low recallers (neutral)	--	--	--	--	Pz: *P* > 0.4 T3: *P* > 0.05	--	--	--
High vs. low recallers (negative)	--	--	--	--	Pz: *T*_(11)_ = 2.33; *P* = 0.040^*^, unc. T3: *T*_(11)_ = 3.84; *P* = 0.003^*^^*^	--	--	--
Emotion × Beck anxiety	(× Laterality) *F*_(2,18)_ = 5.89; *P* = 0.018; η*_p_*^2^ = 0.4	*P* > 0.3	*P* > 0.8	*P* > 0.7	*F*_(1,9)_ = 7.8; *P* = 0.021^*^; η*_p_*^2^ = 0.46	*P* > 0.3	*P* > 0.3	*P* > 0.8
Emotion × Number of arousals	*P* > 0.3	*P* > 0.6	*P* > 0.3	*P* > 0.8	*F*_(1,9)_ = 7.6; *P* = 0.022^*^; η*_p_*^2^ = 0.46	*P* > 0.3	*P* > 0.1	*P* > 0.3
Selected clusters								
Emotion	*P* > 0.5	F3, Fz, F4; (Emotion × laterality): *F*_(1,14)_ = 4.39; *P* = 0.046^*^; η*_p_*^2^ = 0.29; power [1 − β err prob]: 0.56 Post-hoc: F3: *T*_(12)_ = 3.09; *P* = 0.009^*^^*^; power [1 − β err prob]: 0.81 Fz: *T*_(12)_ = 1.94; *P* = 0.076 F4: *T*_(12)_ = 2.35; *P* = 0.03^*^, unc.	*P* > 0.6	*P* > 0.5	T3, Pz, T4; *F*_(1,11)_ = 6.61; *P* = 0.026^*^; η*_p_*^2^ = 0.38; power [1-β err prob]: 0.65	*P* > 0.05	*P* > 0.2	*P* > 0.8

Power estimations are given in relevant effects (Win, time-window [1: 0–200 ms; 2: 200–400 ms; 3: 400–600 ms; 4:600–800 ms]; Unc., uncorrected).

Critically, Emotion effects (NEG vs. NEU) also interacted with State and Time-Window, reflecting that emotion-related modulations of ERPs appeared at earlier latency in NREM. A further ANOVA considering only AWAKE trials for separate time-windows and frontal electrodes only (based on visual inspection of ERP topography), without any covariates, revealed significant Emotion × Laterality effects at F3 after correction, 200–400 ms after sound onset (Table 2), with a similar trend at Fz. No emotion effects were observed over the later time-windows 400–600 or 600–800 ms. In addition, neither effect of Dream recall nor any interaction of this factor with Emotion were observed for the AWAKE state, across all time-windows (Table 2).

Overall, these results indicate that NEG vs. NEU voices during wakefulness elicited larger ERP responses from 200 to 400 ms after onset (Fig. 2), presumably corresponding to an enhanced amplitude of the classic P300 waveform. P300 typically reflects stimulus-driven attention, with a fronto-central distribution in passive tasks where salient stimuli are nontargets (Escera et al. 1998; Jeon and Polich 2001; Dominguez-Borras et al. 2008). Similar modulations at these latencies have been previously reported in wakefulness for emotional vs. neutral voices (Wambacq et al. 2004; Liu et al. 2012; Blume et al. 2017), interpreted as a reflexive capture of attention by emotion. Furthermore, these responses appeared similar across HR and LR groups in wakefulness (Supplementary Fig. 2).

Finally, Beck anxiety scores or arousals showed no significant effects, except for a Laterality × Emotion × Beck anxiety interaction in the 0- to 200-ms window (Table 2), which may reflect general heightening of hemispheric dominance in the initial emotion response for anxious individuals (note that there was no significant Emotion effect in this window).

Focusing next on NREM trials, ERPs exhibited a large negative deflection, with centro-parietal distribution, peaking around 400 ms after stimulus-onset (Fig. 3). This deflection may correspond to the early phase of the N300-N550 complex, typically recorded during the first stages of NREM sleep (see Atienza et al. 2001). The N550 is a frontally distributed component (Perrin et al. 2000; Gora et al. 2001) reflecting the occurrence of K-complexes. K-complexes are large EEG grapho-elements occurring in NREM sleep, linked to mechanisms of protection of sleep, but also to the processing of external stimulation (Wauquier et al. 1995; Halasz 2016). In contrast, the N300 usually shows a central topography (Perrin et al. 2000; Gora et al. 2001) and seems more independent of occurrences of K-complexes (see Colrain et al. 2000). Please note that we did not specifically separate trials containing K-complexes from those where K-complexes did not occur.

ANOVAs on NREM epochs, for separate time-windows and centro-parietal electrodes (based on visual inspection of ERP topography), without any covariates, revealed that Emotion effects mainly arose in the 0- to 200-ms window (Table 2; Fig. 3). In contrast, a more delayed response pattern was observed during AWAKE epochs and accounts for the triple interaction described above (State × Time-Window × Emotion). These findings suggest that, in NREM, emotional voices elicited differential responses with respect to neutral voices around the early phase of the N300 deflection, a component that is known for being particularly sensitive to the meaning of stimuli (Perrin et al. 1999; Perrin et al. 2000) or stimulus salience (Bastien and Campbell 1992) during sleep.

Concerning individual differences, only the analysis of the 0–200 ms-window revealed an Emotion × Dream recall interaction, indicating that differential responses to NEG vs. NEU voices varied between HRs and LRs at this latency (Table 2; Supplementary Fig. 1). A main Emotion effect was observed for HRs and, with a smaller effect size, for LRs as well, with no specific spatial distribution of these effects across groups. Therefore, we selected the midline electrode (Pz) to perform post-hoc independent sample *t*-tests comparing amplitude values across HRs and LRs and found a difference between groups only for the NEG condition, but not for the NEU condition. See also Supplementary Table 1 for additional control analyses using permutation-based *t*-tests.

Additionally, we also examined T3 ad hoc for consistency with our cluster analysis, and for comparability with our time–frequency results (see next section). However, we found a similar trend. In turn, Beck anxiety scores showed a significant interaction with Emotion, selective for the 0- to 200-ms window (Table 2), in agreement with anxiety levels influencing emotion detection (Mogg and Bradley 2002; Rossignol et al. 2012). Furthermore, the occurrence of NREM arousals also interacted with Emotion in this window, an effect further confirmed in subsequent analyses (see NREM arousals section). Importantly, no Emotion effects were noted for the Control sounds when performing similar ANOVAs (Fig. 3), supporting the idea that emotional relevance, not low-level auditory features, explained these effects.

Altogether, these results suggest that HRs had increased stimulus-responsivity during NREM sleep, specifically for emotional voices, around the early phase of the N300 deflection, with stronger effects than those observed in LRs (Supplementary Fig. 1). Further regression analyses confirmed this interpretation, by showing a significant correlation between Dream recall frequency and mean voltage in Pz for the window 0–200 ms, but only for the NEG condition (NEG: *r* = −0.6; *P* = 0.016, power [1 − β err prob]: 0.74; NEU: *r* = 0.05; *P* = 0.44; Fig. 3). See also Supplementary Table 2 for additional confirmatory analyses using permutation-based correlations. For a direct comparison between the NEG and the NEU conditions, the same correlations performed on subtracted values for NEG vs. NEU (indicating the Emotion effect), on Pz and with Dream recall frequency, again revealed a marked negative slope (*r* = −0.6; *P* = 0.016). This supports the idea that neural responsivity to emotion in voices, and not to any auditory stimulus, was directly related to the dream recalling profile of participants. Again, a similar correlation pattern was observed in electrode T3 (NEG: *r* = −0.6; *P* = 0.015; NEU: *r* = −0.33; *P* = 0.1). Moreover, additional analyses (see Supplementary Material) suggested that dream recall effects did not stem from a more general increase of lighter sleep in HRs.

### Brain Oscillations

**Fig. 4:**
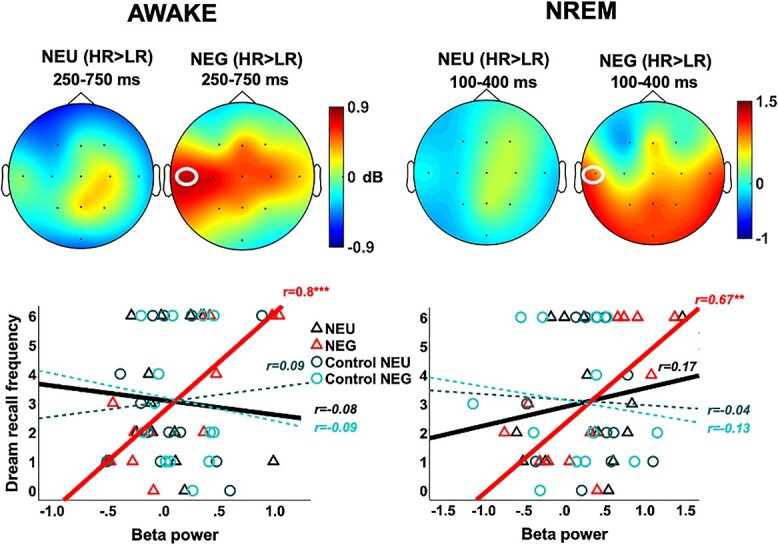
Beta oscillations (18–26 Hz) during wakefulness and NREM. (Top) Scalp distribution plots of beta power differences between high (HR) and low dream recallers (LR), showing significantly increased power in T3 for angry (NEG) voices in HRs. (Bottom) Scatter plots and lines of best fit illustrating beta power for the significant cluster in T3 after each sound condition vs. participants’ dream recall frequency. Beta power after emotional voices increased linearly with individual dream recall. Shaded areas indicate the standard error of the mean (s.e.m.). ^*^^*^P < 0.01; ^*^^*^^*^P < 0.001.

We examined stimulus-related oscillatory responses for the AWAKE and the NREM states separately, with cluster-based permutation tests, using the same 9 channels we considered for the ERP analysis above, and a 0- to 1-s poststimulus window. Only cluster-corrected results are reported. Our first approach directly compared responses to NEG vs. NEU sounds across all participants for AWAKE trials (two-tailed paired *t*-test). This revealed no significant differences in power for any of the frequency-bands, indicating no emotion-related oscillatory responses in wakefulness overall. However, in the comparison of HRs vs. LRs (using independent sample *t*-tests, two-tailed), we did observe a sustained group difference in beta power for the NEG sounds, across centro-lateral sites, reaching significance in electrode T3 from 250 to 750 ms, and for frequencies ranging from 18 to 26 Hz (cum-T = 1946.34; *P* = 0.011; Fig. 4; Supplementary Fig. 3). Such group difference was not observed for the NEU condition. This suggests that beta power may subserve emotion-related responses to NEG vs. NEU voices during wakefulness, but this activity pattern may depend on individual differences associated to brain reactivity. This was supported by further linear regressions, showing that beta power within this cluster correlated strongly with Dream recall frequency across all participants, specifically for the NEG condition (*r* = 0.8; *P* = 0.0005, power [1 − β err prob]: 0.97; NEU: *r* = −0.08; *P* = 0.4; ControlNEU: *r* = 0.09; *P* = 0.4; ControlNEG: *r* = −0.09; *P* = 0.4; Fig. 4; see also Supplementary Table 2 for confirmatory permutation-based analyses). Additionally, the same correlation performed on Dream recall frequency and the beta-power difference from the subtraction of NEG vs. NEU within this cluster (size of Emotion effect) yielded a robust positive slope (*r* = 0.7; *P* = 0.004).

**Fig. 5:**
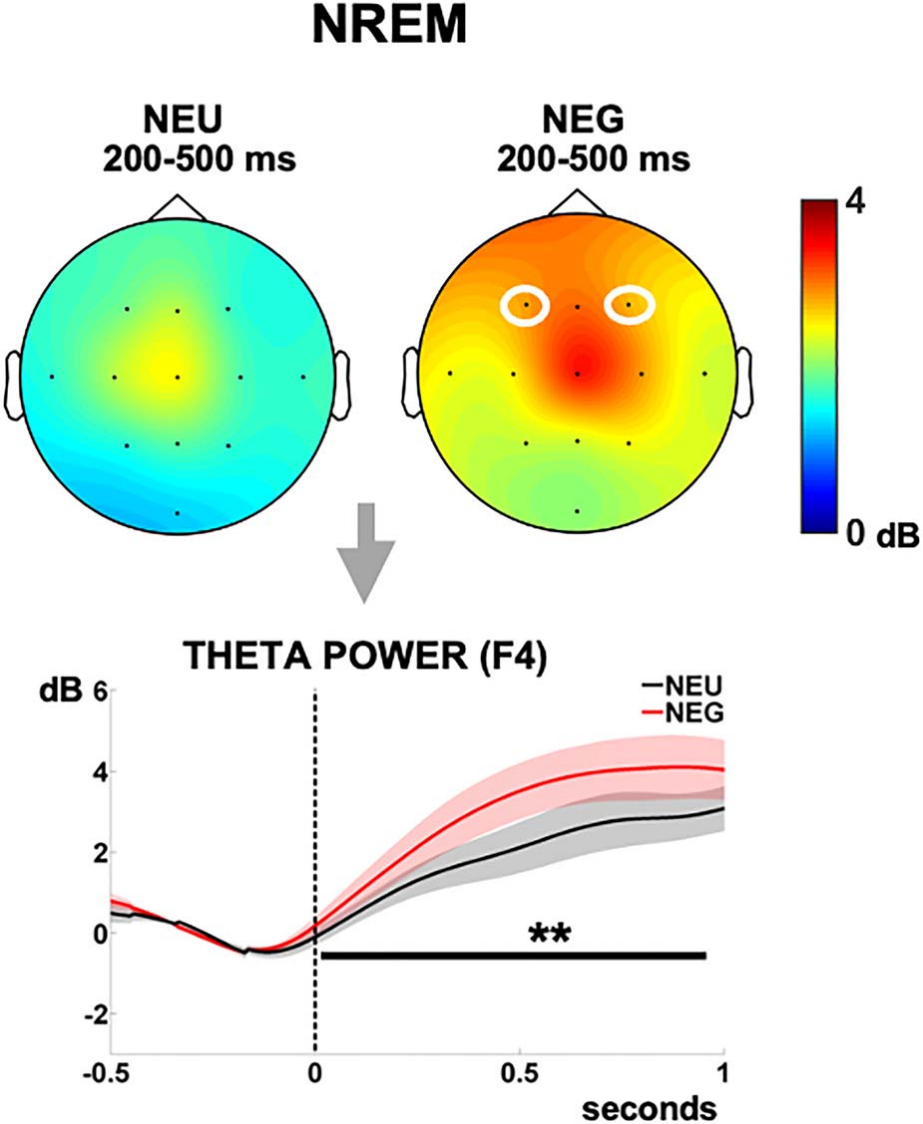
Theta oscillations (4–8 Hz) during NREM. (Top) Scalp distribution plots of theta power for the neutral (NEU) and the angry (NEG) voices, showing significantly increased power in F3 and F4 for NEG. (Bottom) Power time-course for the 4–8 Hz theta cluster in F4, showing significant NEG vs. NEU differences. Shaded areas indicate the standard error of the mean (s.e.m.). ^*^^*^P < 0.01.

During NREM, the comparison NEG vs. NEU across all participants revealed a significant power increase in frontal electrodes (F3 and F4) within the theta band (4–8 Hz), in line with a typical frontal dominance of this frequency band (Bastiaansen et al. 2002). This increase was sustained over the full 1-s window examined (F3: cum-T = 1786.83; *P* = 0.002; F4: cum-T = 1583.57; *P* = 0.002; Fig. 5). Interestingly, no difference between HRs and LRs was seen for this frequency range. Accordingly, theta power values within the cluster peak at F4 (where emotional modulation was largest across both channels) did not correlate with Dream recall frequency for any condition (NEU: *r* = 0.35; *P* = 0.12; NEG: *r* = 0.24; *P* = 0.2; ControlNEU: *r* = 0.03; *P* = 0.5; ControlNEG: *r* = 0.14; *P* = 0.3). This suggests that frontal theta may index emotion processing of sounds during NREM sleep, regardless of individual differences in brain reactivity associated to dream recall.

**Fig. 6:**
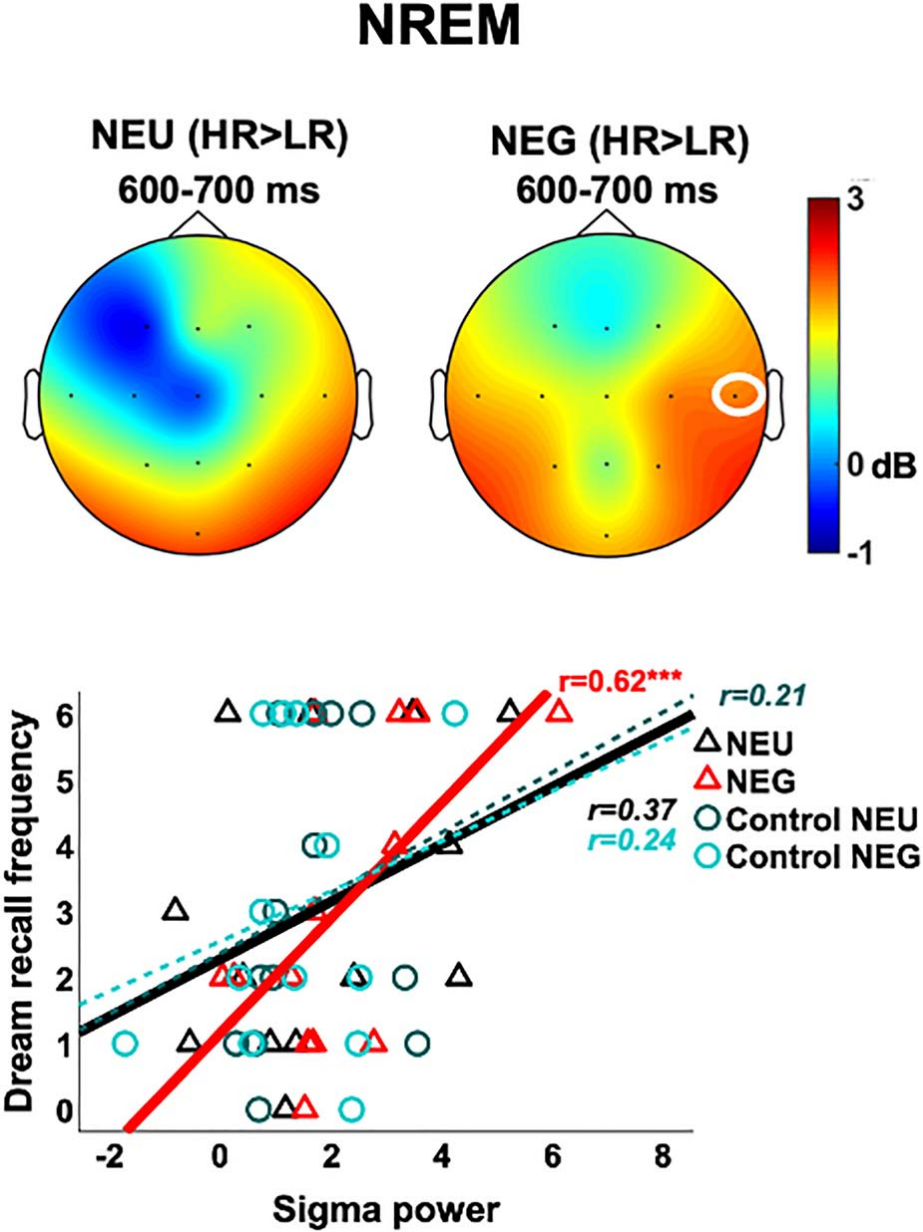
Sigma oscillations (11–15 Hz) during NREM. (Top) Scalp distribution plots of sigma power differences between high (HR) and low dream recallers (LR), showing significantly increased power in T4 for angry (NEG) voices in HRs. (Bottom) Scatter plots and lines of best fit illustrating sigma power for the significant cluster in T4 after each sound condition vs. participants’ dream recall frequency. Sigma power after the emotional voices increased linearly with individual dream recall. ^*^^*^^*^P < 0.001.

In turn, spindle-associated activity after sounds (11–15 Hz) did not show overall emotion effects (NEG vs. NEU across all participants) but yielded a group difference, with sustained increase in power for the NEG condition in HRs vs. LRs during a time-cluster that reached significance from 100- to 1000-ms poststimulus in electrode T4 (11–15 Hz; cum-T = −1681.66; *P* = 0.014; Fig. 6; Supplementary Fig. 3). Even though this effect reached significance for the right hemisphere only, the group difference appeared distributed across bilateral and posterior sites (Fig. 6). Interestingly, and consistent with our results for beta power in AWAKE (above), the spindle difference was not present for the NEU condition. Accordingly, mean sigma power in the peak of this cluster (around 600- to 700-ms poststimulus, where T-values were largest) correlated strongly with Dream recall frequency across all participants, again only for the NEG condition (*r* = 0.62; *P* = 0.012, power [1 − β err prob]: 0.77; NEU: *r* = 0.37; *P* = 0.1; ControlNEU: *r* = 0.21; *P* = 0.2; ControlNEG: *r* = 0.24; *P* = 0.2; Fig. 6; see also Supplementary Table 2). These results suggest that sigma activity, typically associated to spindles (De Gennaro and Ferrara 2003), may also be linked to emotion processing during NREM sleep, but in a way that varies with individual differences in dream recall.

For beta activity, no significant emotion or group effects were observed during NREM when including all channels. We also examined NREM oscillatory responses to sounds in the full 1-s poststimulus window, but in the frequency range (18–26 Hz) and channel (T3) corresponding to the significant beta cluster observed in wakefulness. These analyses did not show NEG vs. NEU differences across all participants, but an HR vs. LR difference for the NEG condition, covering a cluster from 100 to 400 ms (cum-T = 987.33; *P* = 0.01; Fig. 4; Supplementary Fig. 3). Interestingly, and consistently with the AWAKE results, this effect was not significant for the NEU condition. As expected, beta power values within this cluster correlated strongly with Dream recall frequency, only for the NEG condition (*r* = 0.67; *P* = 0.006, power [1 − β err prob]: 0.86; NEU: *r* = 0.17; *P* = 0.3; ControlNEU: *r* = −0.04; *P* = 0.5; ControlNEG: *r* = −0.13; *P* = 0.3; Fig. 4; see also Supplementary Table 2). Similarly, the subtracted (NEG vs. NEU) beta-power values showed a positive correlation with Dream recall frequency (*r* = 0.49; *P* = 0.043). These results suggest that this beta activity component may be independent of consciousness state, as it responds to emotion information during both wakefulness and NREM sleep.

Please note that, despite sigma activity showing similar effects to those observed in beta, we surmise that this activity is genuinely spindle-related and therefore relatively independent from beta. First, the effects on beta were consistently observed above 18 Hz, not below this frequency, indicating that beta (18–26 Hz) and sigma (11–15 Hz) effects did not overlap in frequency. Second, unlike for beta activity, we found no emotion or dream recall effects around the sigma frequency range in wakefulness, even when using less stringent analyses. This suggests that power increases in sigma were selectively produced during NREM, supporting the role of sleep spindles in this effect.

In contrast, neither differences between NEG vs. NEU across all subjects nor between HRs and LRs were observed for the Control sounds when performing similar tests across frequencies of interest, suggesting that differential responses to emotional voices above reflected the processing of the emotional content of sounds, and not of other physical acoustic features. Finally, additional control analyses (see Supplementary material) suggested again that the dream recall effects did not stem from an imbalanced number of trials from the N2 stage in HRs compared with LRs. Please also note that we only report effects on oscillatory responses that survived stringent analyses, but we cannot exclude that other frequency bands may also contribute to affective processing during sleep and/or wakefulness (e.g. delta, Knyazev 2007, 2012; or gamma, Balconi and Pozzoli 2009).

### NREM arousals

**Fig. 7:**
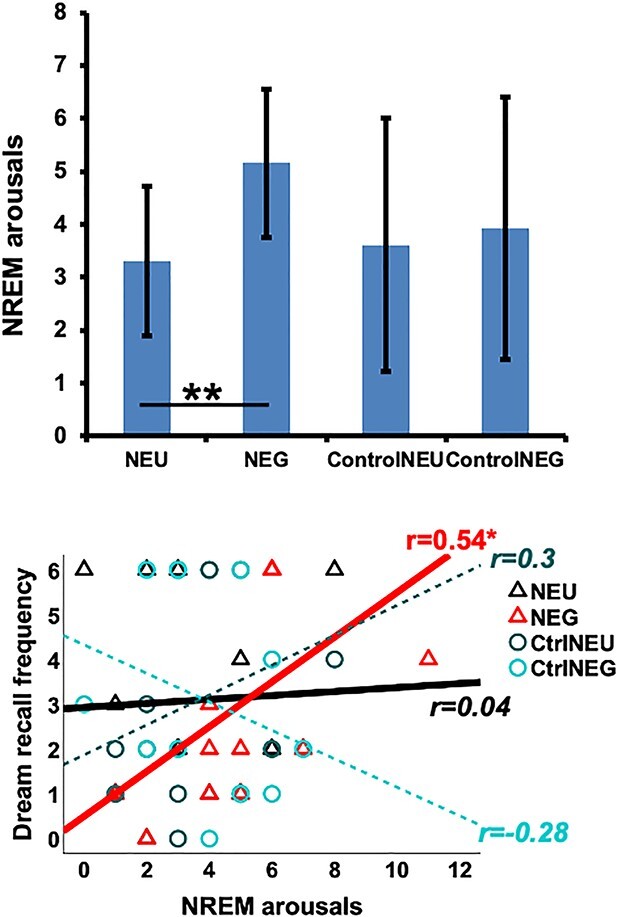
(Top) Number of NREM arousals after each sound condition (±s.e.m.) for all participants. (Bottom) Scatter plots and lines of best fit illustrating the number of arousals during NREM after each sound condition vs. participants’ dream recall frequency. Arousals after the emotional voices increased linearly with dream recall frequency. ^*^P < 0.05.

Arousals during NREM sleep were overall more frequent after NEG voices than after NEU voices (Emotion: *F*_(1,11)=_10.71; *P* = 0.007; η*_p_*^2^ = 0.49; Fig. 7). This effect was specific to real voices (Emotion in NEU vs. NEG: *F*_(1,11)_ = 9.36; *P* = 0.011; η*_p_*^2^ = 0.46) and not observed for control stimuli made of white noise (Emotion in ControlNEU vs. ControlNEG: *P* = 0.9; Fig. 7). Moreover, we found a triple interaction between Stimulus type, Emotion, and Dream recall, suggesting that this effect was amplified in HRs compared with LRs (*F*_(1,11)_ = 5.69; *P* = 0.036; η*_p_*^2^ = 0.34, power [1 − β err prob]: 0.65; Supplementary Fig. 3a). Separate post-hoc pairwise Wilcoxon tests for HRs and LRs revealed a trend to significance when comparing arousals after NEG vs. NEU voices in HRs (Z = 1.76; *P* = 0.078) and a nonsignificant difference for the same analysis in LRs (*P* = 0.24). See also Supplementary Table 1 for additional confirmatory analyses using permutation-based paired tests. Our results suggest that the emotional content of the sounds delivered during NREM sleep played a direct role in the occurrence of arousals during this state, and this effect was more representative of HR than LR individuals. These effects were most likely linked to sound occurrence, given our restrictive analyses, and despite a slight (nonsignificant) difference in sleep patterns between the groups (Table 3; please also note that the proportion of arousals after the sounds was small compared with the total number of arousals during the night). Regression analyses between the number of arousals for each condition and Dream recall frequency confirmed this observation, by revealing a positive correlation (uncorrected) for the NEG condition (*r* = 0.54; *P* = 0.028, power [1 − β err prob]: 0.65), but importantly not for NEU (*r* = 0.044; *P* = 0.4) or for the Control conditions (ControlNEU: *r* = 0.3; *P* = 0.2; ControlNEG: *r* = −0.28; *P* = 0.2; Fig. 7).

**Table 3:** Sleep parameters for high and low dream recallers during the experimental night.

Sleep parameters	All participants mean (±SD)	High recallers mean (±SD)	Low recallers mean (±SD)	Standard
TIB (min)	503 ± 41	507 ± 39	499 ± 48	390–510
SPT (min)	484 ± 35	485 ± 30	484 ± 43	
Wakefulness during TIB (min)	28 ± 19	31 ± 15	25 ± 23	20–30
TST (min)	430 ± 43	421 ± 32	438 ± 55	
Sleep efficiency (%)	86 ± 7	83 ± 5	88 ± 9	80–90
Sleep stage, % of the TIB				
N1 (%)	9 ± 4	11 ± 4	7 ± 5	5–10
N2 (%)	50 ± 6	50 ± 4	50 ± 8	40–55
N3 (%)	20 ± 7	18 ± 4	20 ± 9	25–30
REM sleep (%)	16 ± 3	15 ± 2	17 ± 2	20–25
N1 latency from lights out (min)	6 ± 4	7 ± 4	6 ± 4	
N2 latency from lights out (min)	16 ± 11	21 ± 12	10 ± 6	20–30
REM latency from lights out (min)	117 ± 36	121 ± 11	112 ± 52	
Arousals per hour of sleep	25 ± 7	28 ± 8	23 ± 6	

TIB: time from lights out to lights on. Sleep period time (SPT): time from the beginning of the first episode of sleep to the end of the last episode of sleep. TST: sum of N2, N3, and REM episodes. Sleep efficiency is the TST/SPT. Sleep stages are expressed in percentage relative to the TIB [N1(%), N2(%), N3(%), and REM(%)]. Arousal per hour of sleep refers to sleep stages N2, N3, and REM. Sleep stage 1 (N1), sleep stage 2 (N2), sleep stage 3 (N3), Rapid eye movement sleep (REM); min, minutes; SD, standard deviation of the mean. According to the standard values presented in the last column (Hirshkowitz 2004), sleep quality of participants was generally preserved, with a reduction of N3 and REM sleep, possibly related to the delivery of sounds. No statistical differences in sleep patterns were observed between high and low dream recallers.

Furthermore, the identified arousals started on average 2.1 s after the onset of the preceding sound (see Supplementary material and Supplementary Fig. 3b). Because the arousals followed a stereotyped pattern of occurrence after the preceding stimuli, we surmise that these arousals were stimulus-driven and triggered by the preceding sounds (but not spontaneously generated).

### Sleep parameters

For exploratory purposes, general sleep parameters (relative to standards in general population) were analyzed from EEG recorded overnight. Reliable data could be used from 10 participants (5 HRs and 5 LRs), as 3 participants were excluded due to EEG signal loss during short periods of the night. For all participants, whereas total time in bed (TIB), sleep efficiency, and proportion of lighter N2 sleep were within standards (Hirshkowitz 2004), the total proportion of N3 and REM sleep appeared generally reduced (mean 20% vs. the standard 25–30%, and 26% vs. the standard 20–25%, respectively; Table 3), possibly caused by the delivery of auditory stimuli (Ermis et al. 2010). LRs and HRs did not differ in any of the sleep parameters (independent sample Mann–Whitney U-tests: *U* > 6; *Z* < 1.3; *P* > 0.2). However, slight decreases were apparent in HRs relative to LRs in total sleep time (TST, mean 421 vs. 438 min, respectively), sleep efficiency (mean 83 vs. 88%), proportion of N3 (mean 18 vs. 20%), and REM sleep (mean 15 vs. 17%; Table 3). In turn, N2 and REM latencies appeared increased in HRs, in relation to LRs, although N2 latencies were within the normal range for HRs (mean 21 min vs. the standard 20–30 min) and reduced in LRs (mean 10 min). Similarly, total wakefulness during TIB appeared slightly higher than the normal range in HRs (31 min vs. the standard 20–30 min), whereas it was within standards in LRs (25 min). Although group differences in this parameter were deemed nonsignificant (*U* = 9; *Z* = 0.6; *P* = 0.5), a trend to increase in overall awakenings in HRs may be in agreement with previous reports (Eichenlaub, Bertrand, et al. 2014). Finally, the number of arousals per hour of sleep appeared slightly increased in HRs (28 arousals), relative to LRs (23 arousals), although again with no significant difference (*U* = 7; *Z* = 1; *P* = 0.3).

## Discussion

We examined whether brain reactivity to emotional information persists during NREM sleep as well as during wakefulness, using well-validated voice stimuli with either angry or neutral prosody (Grandjean et al. 2005). We also tested whether such emotional responses depend on individual differences associated to arousal processes and dream recall frequency. First, we show preserved affective auditory processing during stages N2 and N3 of NREM sleep, despite a partial cortical disconnection from external sensory environment in these stages (Jouvet 1967a; Atienza et al. 2001). Our results accord with, and extend, recent EEG findings by showing that a “sentinel processing mode” for emotion detection remains active throughout NREM sleep (Blume et al. 2017, 2018). Second, we show that affective responsivity is strongly influenced by individual traits linked to dream recall. This finding supports the idea that specific mechanisms subserving emotional salience detection during sleep may have an important role in dream memory. Third, we also uncover emotion response patterns that do not vary as a function of individual dream recall, suggesting neural pathways for the detection of emotionally salient/threatening stimuli regardless of individual reactivity. Importantly, our novel results linking dream recall frequency and selective reactivity toward emotional signals in sleep highlight that dream encoding and dream report might be partly rooted in affective brain circuits (Perogamvros and Schwartz 2012). Below we discuss these main results in more details.

### Selective Emotional Responses In NREM Sleep

We observed larger P300 amplitude in frontal electrodes for emotional vs. neutral stimuli during wakefulness, consistent with previous literature suggesting an increased allocation of attentional resources to affectively relevant stimuli (e.g. Wambacq et al. 2004; Delplanque et al. 2006; Dominguez-Borras et al. 2008). In comparison, ERPs during NREM showed an increased negativity at earlier latencies, around the initial N300. This negativity is also sensitive to stimulus salience (Bastien and Campbell 1992) and stimulus meaning during NREM sleep (Perrin et al. 1999, 2000). Alternatively, an increased negativity around N300 might result a reduction of the preceding P200 component for emotional vs. neutral voices. Previous research suggested that P200 during N3 sleep may reflect reactive cortical inhibition, preventing further stimulus processing (Andrillon et al. 2016). Accordingly, a reduced P200 would imply weaker inhibition and, therefore, enhanced processing of emotional stimuli.

Simultaneously, we observed a selective emotional modulation of oscillatory theta responses in frontal regions during NREM. Frontal theta in NREM has been linked to memory, reflecting hippocampo-cortical feedback loops (Klimesch 1999). However, more generally, theta is also strongly associated to emotional arousal (Sainsbury and Montoya 1984; Nishitani 2003; Knyazev 2007), orienting toward emotional faces or scenes (Krause et al. 2000; Knyazev 2007; Balconi and Pozzoli 2009), as well as electrodermal responses to emotional stimuli (Aftanas et al. 2004). In fact, theta may partly be generated in the amygdala (Knyazev 2007; Maratos et al. 2009), where neurons show higher theta oscillations during emotional arousal (Pare and Collins 2000; Pare 2003; DeJean et al. 2015; Karalis et al. 2016). Importantly, we did not observe similar responses for control auditory stimuli with similar energy envelopes as the emotional voices (Banse and Scherer 1996; Grandjean et al. 2005), indicating that these effects were driven by the affective significance of stimuli, not by acoustic features.

Finally, and importantly, arousals recorded during NREM sleep were more frequent after angry than neutral voices (again with no such difference for control voices), confirming that emotional information increased alertness and thus triggered more frequent subtle awakenings (Iber et al. 2007). This extends previous research reporting similar effects after nociceptive stimuli in sleep (Bastuji et al. 2008) and confirms that sleep arousals provide a reliable measure of transient alertness evoked by external salient stimulation (Vallat et al. 2017).

### Emotion Responses Modulated By Individual Dream Recall Propensity

Several dimensions of emotional responses observed during NREM correlated with individual dream recall frequency, a behavioral trait previously associated to cortical reactivity during sleep (Ruby et al. 2013b; Eichenlaub, Bertrand, et al. 2014; Eichenlaub, Nicolas, et al. 2014; Vallat et al. 2017). First, amplitude of ERPs after emotional voices during NREM increased linearly with individual dream recall rates, especially within the first 200-ms poststimulus. This correlation was absent for neutral voices or for emotional voices during wakefulness. Secondly, beta oscillations, as well as spindle-associated sigma activity after emotional stimuli, were also associated with dream recall (unlike theta oscillations). Even though beta oscillations remain poorly understood (Engel and Fries 2010; Davis et al. 2012), they were already considered a marker of emotional processes in prior research (Ray and Cole 1985). Similar increases in evoked beta during wakefulness have been reported for emotionally negative facial expressions (Guntekin and Basar 2007) and negative pictures (Guntekin and Basar 2010), relative to positive or neutral stimuli. More generally, beta activity predicts perception accuracy (Gail et al. 2004; Wilke et al. 2006; Hipp et al. 2011) and correlates with attention in early sensory (visual) cortex (Siegel et al. 2008). Altogether, this suggests that beta activity may index overall sensitivity to negative emotional signals (Guntekin and Basar 2010).

In addition, we found that spindle-related responses (i.e. sigma power) in NREM exhibited a similar pattern, with selective increases in power after emotional voices that varied linearly with dream recall frequency. Spindles play a critical role in memory consolidation and plasticity in NREM (Born and Wilhelm 2012; Rasch and Born 2013; Staresina et al. 2015), and higher spindle activity may reflect off-line reprocessing of emotionally relevant memories (Igloi et al. 2015; Lehmann et al. 2016). Conversely, spindle oscillations have also been linked to the inhibition of thalamocortical transmission of incoming sensory information, for protection of sleep (Cote et al. 2000; Sato et al. 2007; Schabus et al. 2012), and their presence predicts attenuated cortical responses to sounds (Elton et al. 1997; Dang-Vu et al. 2011; Schabus et al. 2012). Previous investigation in N2 sleep described similar increases in sigma power after emotional voices, compared with neutral, and concluded that this may reflect persistent effort for inhibition of sensory processing (Blume et al. 2017). Finally, while the number of arousals during sleep increased after the presentation of emotional voices, it also correlated significantly with dream recall frequency reported by participants. Again, this was not true for arousals following neutral or control stimuli. This finding clearly supports the idea that individual dream recall may be a reliable marker of salience/affective reactivity in sleep, rather than more generalized alertness toward sensory inputs.

### Dream Recall Is Selectively Determined By Emotion Reactivity

All reported correlations with dream recall frequency were observed for emotional voices, and generally not for neutral or control stimuli. This converges with the idea that the generation and/or memory for dreams is closely related to the activation of limbic/mesolimbic brain circuits, which may thus explain an abundance of emotionally salient information in high dream recallers (see Perogamvros and Schwartz 2012, 2015; for reviews). Other studies reported that high compared with low recallers are more responsive to novel stimuli (Ruby et al. 2013a, 2013b;Eichenlaub, Bertrand, et al. 2014 ; Vallat et al. 2017). High recallers may thus present enhanced excitability of affective or saliency networks, which may in turn lead to a selective lowering of arousal thresholds for emotional stimulation. This would then provide optimal conditions for the encoding, and/or subsequent access to, dream memories. Indeed, the “arousal-retrieval” model (Koulack and Goodenough 1976) postulates that dream encoding in long-term memory, and subsequent dream recall, depends critically on intrasleep arousals or awakenings that favor long-term retention in memory. Here, we further suggest that individual salience reactivity is critical in the occurrence of such sleep perturbations. It has also been suggested that high recallers may have increased production of dreams (Eichenlaub, Bertrand, et al. 2014). However, our results cannot help dissociate dream recall from dream production, given the well-known methodological difficulty to implement such distinction in dream research (Schwartz and Maquet 2002; Desseilles et al. 2011).

Notably, emotional biases in high recallers may not be associated to other individual psychological traits that are known to affect emotion reactivity, such as anxiety (Rossignol et al. 2012). Although, as expected, we found that Beck anxiety scores did interact with specific emotion effects in the ERPs, none of the scores obtained in our questionnaires, including anxiety, correlated with dream recall frequency. Other psychological traits associated with high recallers, such as openness to experience and absorption (Schredl et al. 2003), might be related to differences in emotion reactivity, but these were not evaluated here.

A link between dream recall and individual emotional biases is also reminiscent of the fact that dreams are often reported with high emotional content in the overall population (Desseilles et al. 2011; Ruby 2011). Moreover, emotion intensity in dreams depends on amygdala function (Blake et al. 2019). Although dreaming has been more often studied in REM sleep, NREM is also rich in oniric activity (Desseilles et al. 2011; Siclari et al. 2013, 2017) and shows increased metabolism in limbic regions compared with wakefulness (Nofzinger et al. 2002). Even though there is no specific evidence for high recallers having stronger basal activity in these regions, a differential recruitment of emotion systems may partly explain why the brain remains more emotionally reactive in people who remember best their dreams. Our results in NREM therefore accord with the idea that stimulus–response patterns in this state may be highly informative for understanding the dreaming brain (Solms 2000; Eichenlaub, Bertrand, et al. 2014). Finally, we note that dream recall effects were observed despite both groups showing no significant differences in basic sleep parameters throughout the night, in agreement with previous reports (Eichenlaub, Bertrand, et al. 2014; Vallat et al. 2017). Furthermore, all our effects were observed regardless of the proportion of N2 vs. N3 trials in individual data, indicating that dream recall results did not depend on sleep depth.

In sum, our results underline the important role of emotion biases in memory for dreams. This suggests that the individual ability to recall dreams is intimately connected with selective reactivity to affectively salient inputs, and not to generalized brain responsiveness to external sensory stimulation (Ruby et al. 2013a, 2013b; Eichenlaub, Bertrand, et al. 2014; Vallat et al. 2017).

### Theta Response to Emotion in NREM is Independent of Individual Dream Recall

Although this may be speculative and demands further research, we suggest that theta responses could constitute an oscillatory signature of automatic limbic responses toward emotional information (Tamietto and de Gelder 2010; Dominguez-Borras et al. 2012), as it remained invariant despite brain reactivity reflected in dream recall. Similar early and prolonged (0- to 600-ms poststimulus) increases in theta power have been reported for emotional vs. neutral pictures in scalp-EEG (Aftanas et al. 2004), suggesting that emotion encoding is associated with fast and sustained theta response (Aftanas et al. 2001; Symons et al. 2016). Accordingly, amygdala oscillatory response to emotion at very early latencies (40–140 ms) has been previously suggested to reflect an “automatic,” or awareness-independent, process within emotion encoding during wakefulness (Luo et al. 2010). Even though the latter study highlighted gamma activity rather than theta, our early theta responses may similarly reflect a relatively “automatic” and reflexive stage of emotion processing, persisting during NREM.

### Limitations and Open Issues

A first potential limitation of our study is the small sample of participants who completed the full protocol (13 subjects). This was due to the rigorous and demanding experimental setup necessary to ensure optimal sleep and reliable EEG conditions. However, we report effect size throughout, and all main results appeared large enough for reliable interpretation. Moreover, additional permutation-based tests, more suitable for small sample sizes, are reported for all main effects in the Supplementary material. A second limitation is that we merged data from N2 and N3 sleep stages, due to insufficient trials in either stage alone. Although we found that the proportion of trials from either stage could not explain our effects, further research should examine whether emotion reactivity varies across the different sleep stages. Likewise, due to insufficient clean data during REM sleep, we excluded this stage from analysis. However, REM has long been associated with dreaming and emotional processing, given a well-established hyperactivation of emotion-related regions during this stage (Maquet et al. 1996; Braun et al. 1997; Nofzinger et al. 1997). Thus, future research should examine whether affective responsivity also extends in REM sleep and whether it varies similarly with dream recall. Another potential limitation relates to the fact that our data showed consistent correlations between dream recall frequency and responses to emotional but not neutral or control voice stimuli, with one exception: ERP voltage around the first 200-ms postsound for the control neutral condition was also correlated with dream recall during NREM sleep. Such correlation was nevertheless absent for the control version of emotional voices, suggesting that this isolated effect may be a spurious finding. In general, our data clearly converged to suggest that dream recall frequency correlated primarily with emotion-related responses.

## Conclusion

In sum, we demonstrate that emotion reactivity toward external stimulation persists in NREM sleep but depends on individual responsiveness associated to dream recall. We also dissociate frequency components associated with this individual variable (beta and sigma activity) from those that remain invariant (theta activity). Our findings not only characterize residual emotion reactivity during sleep but also shed new light on the debated question of whether emotion detection requires conscious vigilance or still unfolds automatically in some conditions with altered states of consciousness. Finally, our results extend current models of dreaming in humans, by stressing the role of emotion reactivity in memory encoding and/or subsequent recall of dreams.

## Supplementary Material

Moyne_etal_2022_CerCortexComms_SuppMaterial_tgac003
