# Supplementary material for: Brain reactivity to emotion persists in NREM sleep and is associated with individual dream recall
Source: Cereb Cortex Commun. 2022 Jan 27;3(1):tgac003. doi: 10.1093/texcom/tgac003 (PMC8844542; doi:10.1093/texcom/tgac003)
Supplement: Moyne_etal_2022_CerCortexComms_SuppMaterial_tgac003 [file moyne_etal_2022_cercortexcomms_suppmaterial_tgac003.docx]

**Supplementary Material**

**Control analyses for potential confounds relative to number of trials**

***N2 versus N3***

To control for potential confounds derived from an unequal proportion of N2 vs. N3 trials between HRs and LRs in NREM, we compared the number of trials included in the data with a repeated-measures ANOVA, including the factors Stage (N2, N3), Emotion (NEU, NEG), Sound type (Normal sound, Control sound), and the between-subject factor Dream recall (HR, LR). We found no main effects of Dream recall (p=0.19) but an interaction Stage x Dream recall (F_(1, 11)_= 5.14; p=0.045), which stemmed from overall larger number of N2 trials in HRs (M=71.2; SD=25) compared to LRs (M=35.34; SD=20.7; Dream recall effect in N2: F_(1, 11)_= 8.44; p=0.014). Importantly, no interactions of Dream recall were observed with Emotion (p=0.86) or Sound type (Dream recall x Sound type: p=0.14; Dream recall x Sound type x Emotion p=0.87) in this stage, supporting the idea that any emotion-related differences observed across groups may not stem from lighter sleep in HRs. In turn, no group effects (Dream recall: p=0.35), nor interactions thereof (p>0.2), were observed for stage N3, reflecting similar proportion of deep sleep N3 trials across HRs (M=31.17; SD=17.3) and LRs (M=45.53; SD=29.5).

Nevertheless, we still considered relevant to control for number of trials whenever significant Dream recall effects were observed and ran additional linear regressions (two-tailed) between number of N2 trials and our data in each condition of interest. Regarding the ERPs, no significant correlations were observed between the number of N2 trials across all subjects and Pz mean voltage values where main effects were observed (NEU: r=-0.19; p=0.5; NEG: r=-0.09; p=0.8; ControlNEU: r=0.23; p=0.5; ControlNEG: r=0.29; p=0.3). Similarly, for oscillatory activity, no significant correlations were observed between number of N2 trials and mean power values from main significant clusters showing Dream recall effects. This was true for both sigma (NEU: r= 0.1; p=0.7; NEG: r=0.2; p=0.5; ControlNEU: r=0.06; p=0.9; ControlNEG: r=0.2; p=0.5) and beta power (NEU: r=-0.3; p=0.3; NEG: r=0.45; p=0.12; ControlNEU: r=0.07; p=0.8; ControlNEG: r=-0.14; p=0.7). Overall, this indicates that the dream recall effects did not stem from a larger proportion of lighter sleep in HRs.

***Emotional versus neutral voices***

To test whether the emotion effects could originate in different proportion of trials averaged across conditions, we compared the number of trials for all conditions with a repeated-measures ANOVA, including the factors Stage (Awake, NREM), Emotion (NEU, NEG), Sound type (Normal sound, Control sound), and the between-subject factor Dream recall (HR, LR). This revealed no main Emotion effects (p=0.46), but it did show a significant Sound type x Emotion interaction, which originated in a larger proportion of trials for the NEG versus the NEU conditions for NREM (p=0.012), but the opposite for Awake (i.e. a smaller proportion of trials for NEG versus NEU for Awake; p=0.006). Importantly, no interactions of Emotion with Dream recall were observed in any of the tests (p>0.4), supporting the idea that any emotion-related differences observed across groups did not originate in different number of trials among conditions.

In any case, to further control for any possible confounds derived from the observed NEG vs. NEU differences in the number of trials, we performed additional linear regressions (two-tailed) between our voltage values for NEG minus NEU in main electrodes of interest -F3 for Awake, Pz for NREM- for the NEG minus NEU conditions), with the exact corresponding differences in the number of trials (i.e. number of trials in NEG minus number of trials in NEU both for Awake and NREM). These new analyses revealed no significant correlations (p>0.3), again suggesting that our main effects did not stem from a different proportion of trials in the NEU versus the NEG conditions, either for Awake or NREM.

**Control analyses using permutation-based statistics**

For completeness, we performed additional permutation-based analyses (1000 permutations) to confirm our main t-tests and correlations, as these analyses allow for minimal assumptions regarding variables and are thus more desirable with smaller sample sizes. First, we compared our ERP amplitudes (where main emotion effects were observed in Pz) and number of arousals during NREM for high versus low dream recallers (Supp.Table 1). These confirmed the differences among groups in the critical conditions (Negative) despite small sample size.

**Supp.Table 1.** Permutation-based t-tests (T / p-values) of main neurophysiological reactivity measures.

|  | | **NREM** | |
| --- | --- | --- | --- |
|  |  | **Number of arousals** | **ERP amplitude (0-200ms)** |
| **High vs Low**  **dream recallers** | **Neutral** | -0.48/0.33 | 0.1/0.23 |
|  | **Negative** | -3/0.002** | 0.38/0.022* |
|  | **Control neutral** | -1.93/0.050 | 0.11/0.11 |
|  | **Control negative** | 0.2/0.55 | 0.06/0.33 |
| **Negative vs Neutral** | **High recallers** | -3.4/0.048* | 0.27/0.040* |
|  | **Low recallers** | -0.88/0.16 | -0.004/0.49 |
| **Control negative vs Control neutral** | **High recallers** | 1/0.72 | 0.007/0.46 |
|  | **Low recallers** | -1.13/0.11 | 0.06/0.17 |

Moreover, given that correlations with Dream recall frequency were among the most critical results in the study (computed over the whole sample of 13 subjects), we performed additional permutation-based Pearson’s correlations on all our main effects indicated in the text (2-tailed; Supp.Table 2). These results were fully consistent with those (non-permutation-based) reported in the main text. In summary, all signatures of neurophysiological reactivity to the emotional stimuli (except theta, see Discussion) correlated with individual dream recall frequency. Again, this was not true for the neutral or control stimuli. Finally, please note that other results reported in the main text (i.e. time-frequency analyses) were already obtained with permutation tests.

**Supp.Table 2.** Permutation-based correlations of neurophysiological reactivity measures with Dream recall frequency.

| **Correlation coefficient /**  **p-values** | **Awake** | **NREM** | | | |
| --- | --- | --- | --- | --- | --- |
|  | **Beta power** | **Number of arousals** | **ERP amplitude**  **(0-200ms)** | **Beta power** | **Sigma power** |
| **Neutral** | -0.08/0.89 | 0.04/0.86 | 0.049/0.86 | 0.17/0.58 | 0.37/0.18 |
| **Negative** | 0.8/0.012* | 0.54/0.042* | -0.6/0.022* | 0.67/0.010* | 0.62/0.022* |
| **Control neutral** | 0.09/0.79 | 0.3/0.32 | -0.48/0.11 | -0.04/0.9 | 0.21/0.47 |
| **Control negative** | -0.09/0.74 | -0.28/0.35 | -0.08/ 0.77 | -0.13/0.64 | 0.24/0.42 |

**Onset times of NREM arousals after sound occurrence**

To better assess the pattern of occurrence of NREM arousals after the sounds, we compared arousal onset times with respect to the onset of the preceding sound across conditions, by means of a repeated-measure ANOVA. This included the within-subject factors Emotion (NEG vs. NEU) and Sound type (Normal sound, Control sound), and the between-subject factor Dream recall (HR, LR). The results revealed no significant differences among conditions or groups, indicating no major divergences in the delay of their occurrence after the different stimuli.

Additionally, we computed frequency histograms (Supp.Fig. 4) depicting the overall distribution of arousal onset values for each condition, and assessed the fit of these values to a normal distribution for each condition separately with Shapiro-Wilk normality tests (which are especially recommended for sample sizes smaller than 50). These suggested that arousal onset times followed a normal distribution for all stimulus-types (NEG: p=0.19; NEU: p=0.15; ControlNEU: p=0.11; ControlNEG: p=0.093). Note that the null hypothesis of the latter test is that the data are normally distributed, and therefore non-significant p-values indicate normal distribution.

**
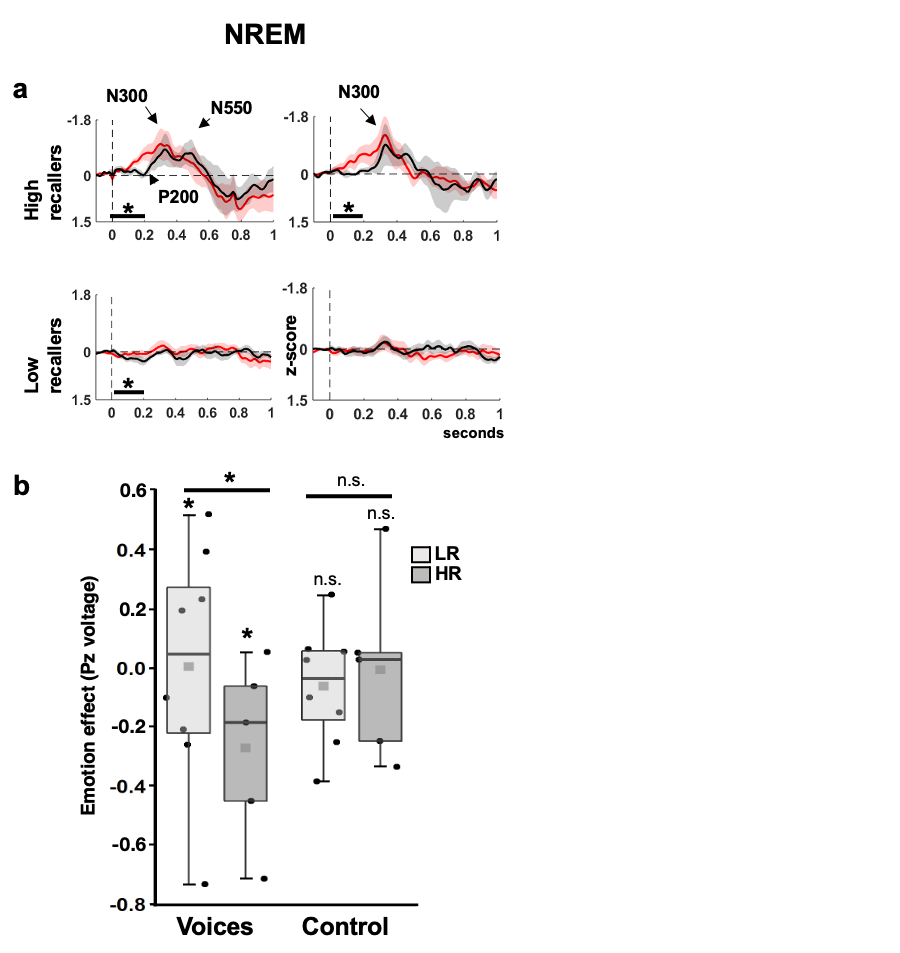
**

***Supp. Figure 1.*** *(a) ERPs to neutral (NEU) and angry (NEG) voices for high and low dream recallers during NREM. Shaded areas indicate the standard error of the mean (s.e.m.). (b) Box plots of amplitude values, averaged from the 0-200ms time-window, depicting emotion differences (NEG minus NEU) for both the normal voices and the control sounds. Grey squares indicate mean values across subjects. Emotional modulation with normal voices was more prominent in high than low recallers, and absent in both groups for control sounds. *p<0.05; n.s., not significant.*

**
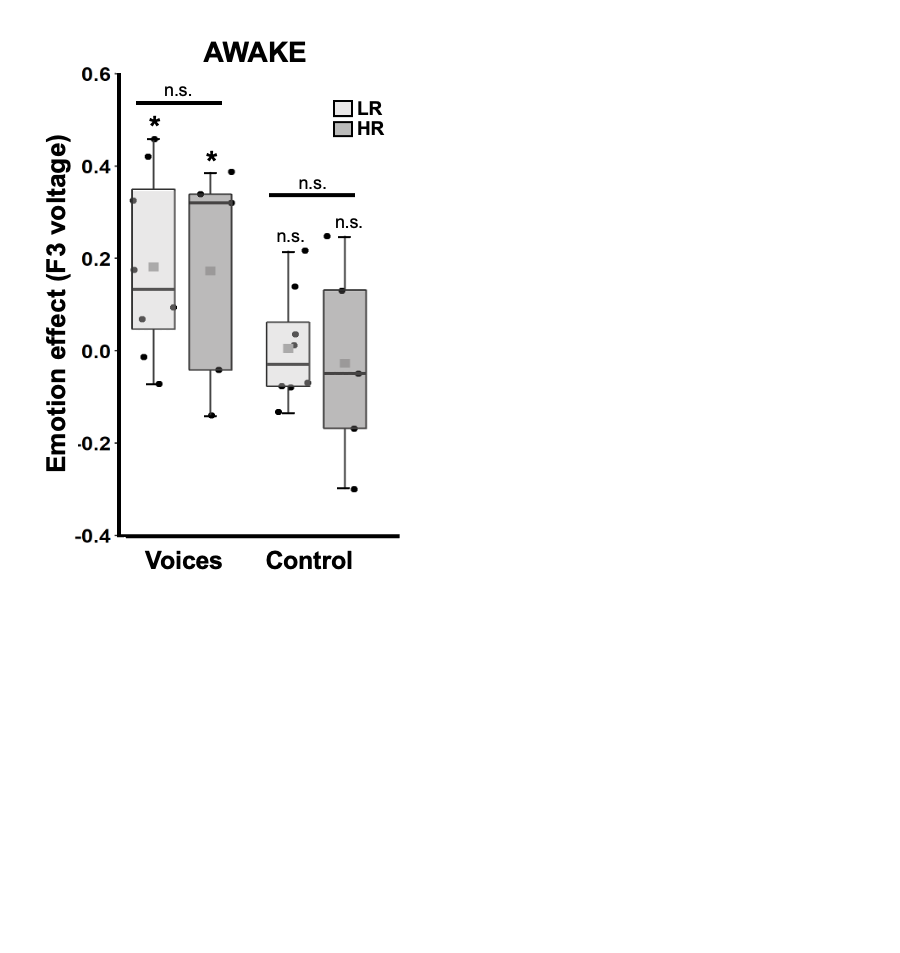
**

***Supp. Figure 2.*** *Box plots of ERP amplitude values, averaged from the 200-400ms time-window, depicting emotion differences (NEG minus NEU) for both the normal voices and the control sounds during wakefulness. Grey squares indicate mean values across subjects. Emotional modulation with normal voices was similar for both groups, and absent for control sounds. *p<0.05; n.s., not significant.*

**
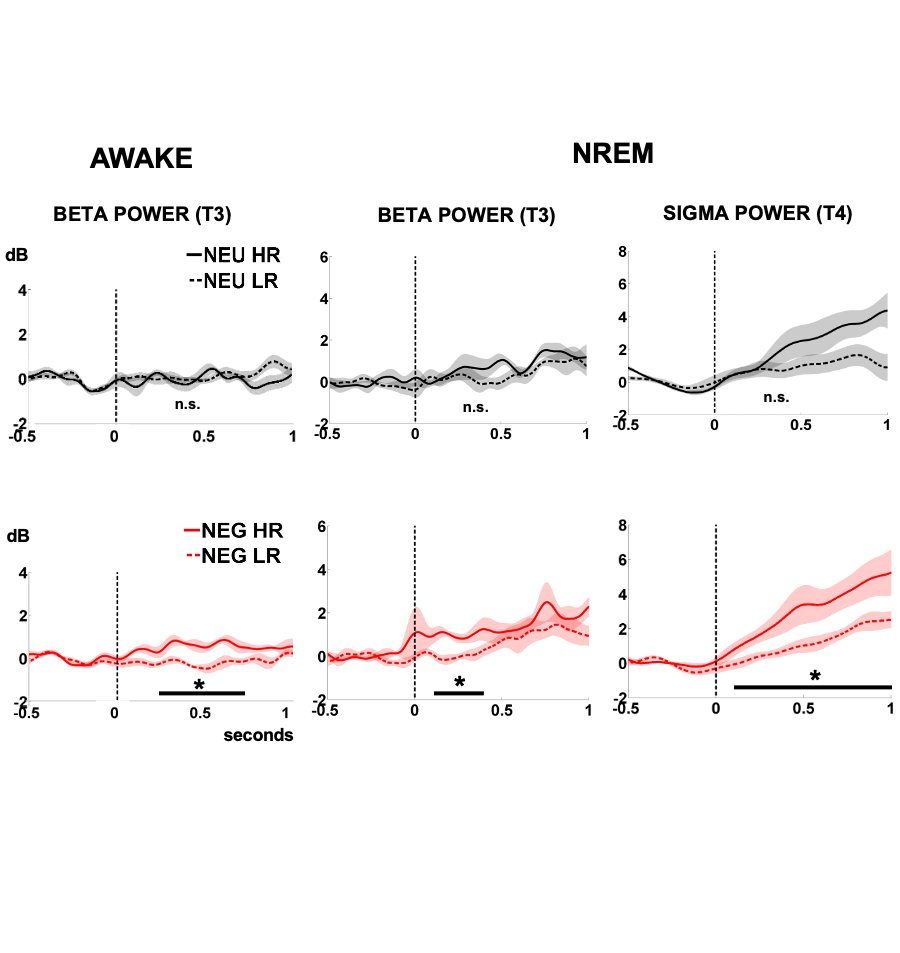
**

***Supp. Figure 3.*** *Power time-courses for the 18-26 Hz beta clusters in T3 for during wakefulness (left) and NREM (middle), and for the 11-15 Hz sigma cluster in T4 during NREM (right). For all three frequency bands, significant HR vs. LR differences were observed for angry voices (NEG), but not for neutral (NEU) voices. Shaded areas indicate the standard error of the mean (s.e.m.). *p<0.05.*

**
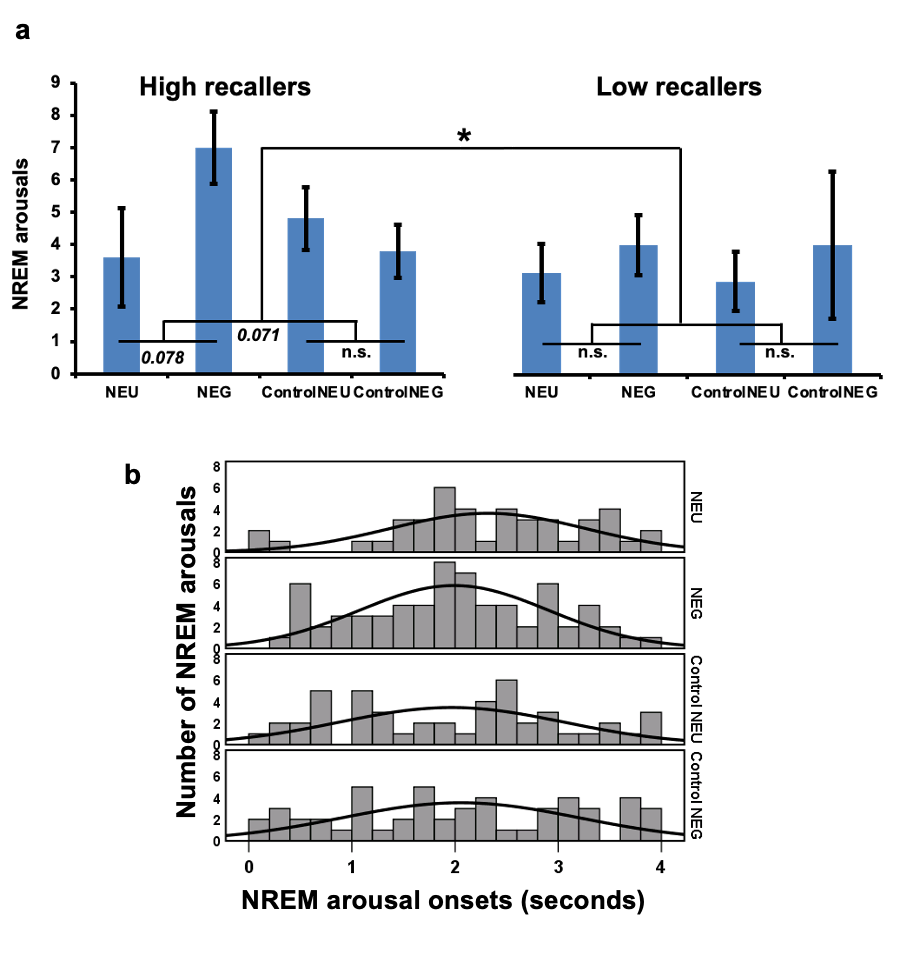
**

***Supp. Figure 4.*** *(a)* *Number of NREM arousals after each sound condition (±s.e.m.) for high and low dream recallers. Arousals were more frequent after angry voices (NEG) than neutral (NEU) or control stimuli, an effect that was predominant in high dream recallers. (b) Frequency histograms and normal distribution fits displaying the onset times of NREM arousals for all participants, after each sound condition. Arousal onset times followed a normal distribution across conditions, indicating that arousals were not random sleep perturbations, and were plausibly triggered by the immediately preceding sounds.*
